# Pitch Class and Envelope Effects in the Tritone Paradox Are Mediated by Differently Pronounced Frequency Preference Regions

**DOI:** 10.3389/fpsyg.2018.01590

**Published:** 2018-09-28

**Authors:** Stephanie Malek

**Affiliations:** Psychology Department, Martin Luther University Halle-Wittenberg, Halle, Germany

**Keywords:** Shepard tones, tritone paradox, psychoacoustics, probabilistic models, pitch perception

## Abstract

Shepard tones (octave complex tones) are well defined in pitch chroma but are ambiguous in pitch height. Pitch direction judgments of Shepard tones depend on the clockwise distance of the pitch classes on the pitch class circle, indicating the proximity principle in auditory perception. The tritone paradox emerges when two Shepard tones that form a tritone interval are presented successively. In this case, no proximity cue is available and judgments depend on the first tone and vary from person to person. A common explanation for the tritone paradox is the assumption of a specific pitch class comparison mechanism based on a pitch class template that is differently orientated from person to person. In contrast, psychoacoustic approaches (e.g., the Terhardt virtual pitch theory) explain it with common pitch-processing mechanisms. The present paper proposes a probabilistic threshold model, which estimates Shepard tone pitch height by a probabilistic fundamental frequency extraction. In the first processing stage, only those frequency components whose amplitudes are above specific randomly distributed threshold values are selected for further processing, and whose expected values are determined by a threshold function. The lowest of these nonfiltered components is dedicated to the pitch height. The model is designed for tone pairs and provides occurrence probabilities for descending judgments. In a pitch-matching pretest, 12 Shepard tones (generated under a cosine envelope centered at 261 Hz) were compared to pure tones, whose frequencies were adjusted by an up-down staircase method. Matched frequencies corresponded to frequency components but were ambiguous in octave position. In order to test the model, Shepard tones were generated under six cosine envelopes centered over a wide frequency range (65.41, 261, 370, 440, 523.25, 1244.51 Hz). The model predicted pitch class effects and envelope effects. Steep threshold functions caused pronounced pitch class, whereas flat threshold functions caused pronounced envelope effects. The model provides an alternative explanation to the pitch class template theory and serves as a psychoacoustic framework for the perception of Shepard tones.

## 1. Introduction

**Figure 1:**
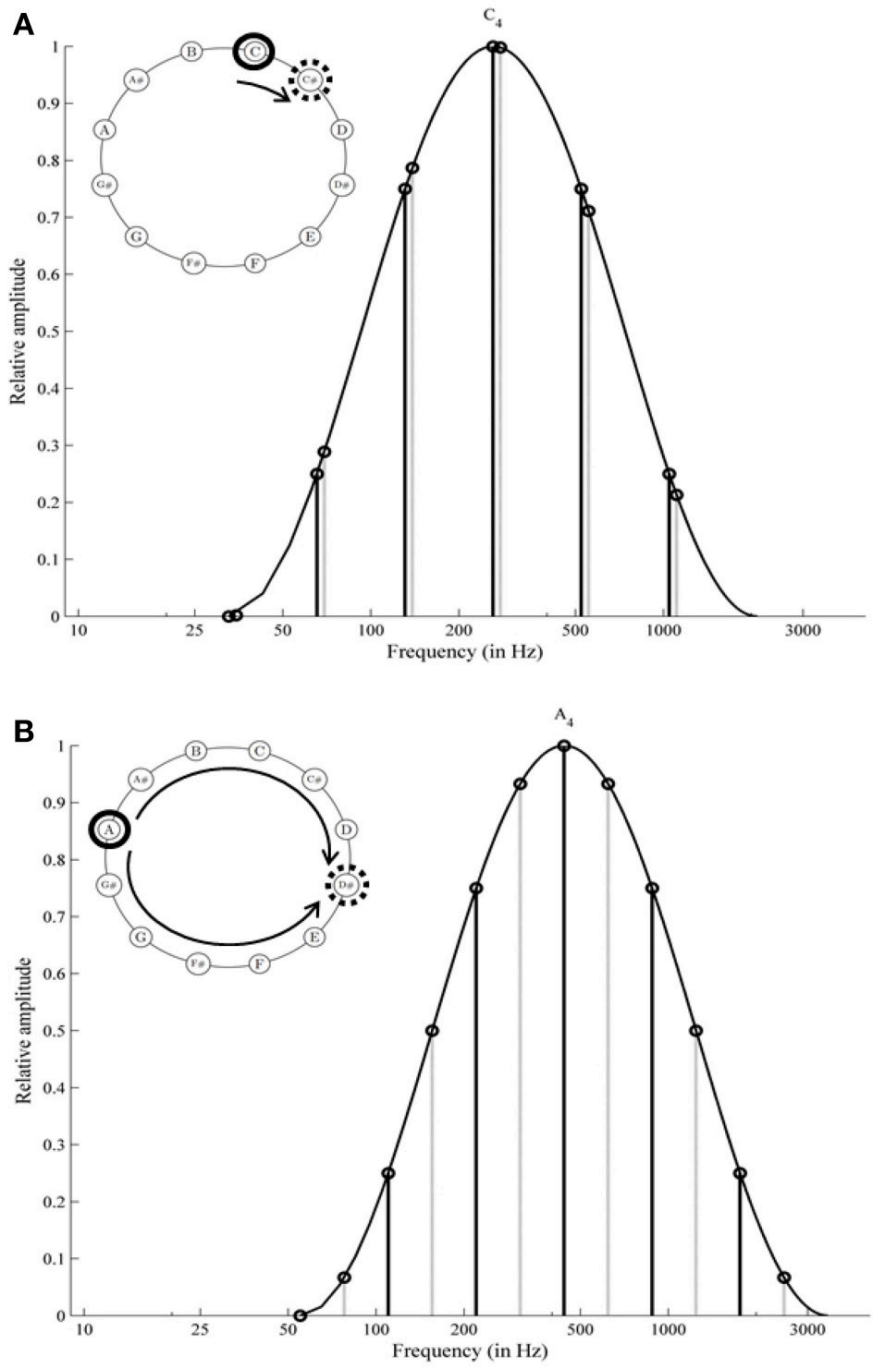
Spectral structure of Shepard tones: *C* and *C#* are generated under an envelope centered at *C*_4_ (261.63 Hz); the components of *C#* are shifted 1 semitone from components of *C*; the distance on the pitch class circle is shorter clockwise than counterclockwise; the frequency shift to the right is shorter than to the left **(A)**; *A* and *D#* are generated under an envelope centered at *A*_4_ (440 Hz); the components of *D#* are shifted 6 semitones from components of *A*; the distance on the pitch class circle is equal clockwise and counterclockwise (tritone interval, **B**). The envelope centered at *A*_4_ **(B)** is slightly shifted to the right in comparison with the envelope centered at *C*_4_ **(A)**. The relation of component amplitudes of *C* under *C*_4_ is the same as the relation of component amplitudes of *A* under *A*_4_.

Shepard tones or octave complex tones evoke some astonishing auditory illusions. These tones consist of several sinusoidal components spaced by octave intervals. Typically, the component amplitude is determined by a fixed bell-shaped spectral envelope over the logarithmic frequency axis. Shepard tones are constructed by equal-sized upward shifts of their components under this fixed envelope (see Figure 1A). Surprisingly, when participants listen to such stepwise increased Shepard tones several times successively, they are normally unaware of any repetitions and report a continuous ascending pitch or a continuous descending pitch for a sequence in reversed order (never-ending pitch illusion; Shepard, 1964; Burns, 1981). Shepard (1964) found that pitch direction judgments of Shepard tone pairs (ascending/descending) depend on the clockwise distance of their pitch classes on the pitch class circle. Specifically, participants judged the pitch direction of Shepard tone pairs as ascending when this distance was shorter clockwise than counterclockwise (see Figure 1A) and descending in the opposite condition. This finding has been replicated by several studies (Pollack, 1978; Sugiyama and Ohgushi, 1979; Burns, 1981) and provides evidence for the proximity principle in auditory perception.

**Figure 2:**
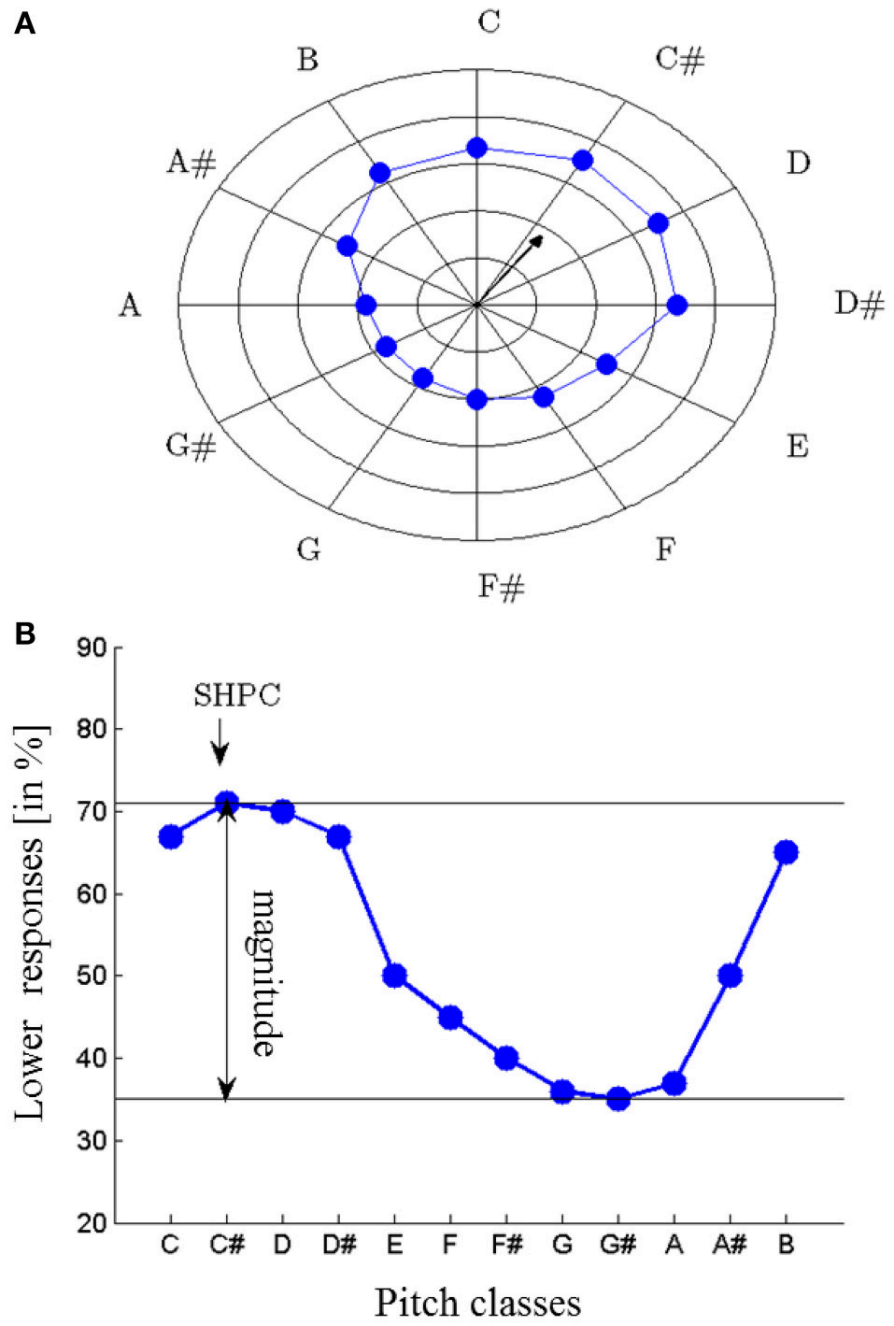
Subjectively highest pitch class (SHPC) and magnitude of effect are extracted from the proportion of *lower* judgments as a function of the initial pitch classes of tritone pairs; plotted in a radial response graph (concentric circles: increments of 20%, **A**) and as response function **(B)**; the SHPC is given by the direction of the resultant vector of the data points **(A)**; for more details (see Fisher, 1993) and is nearly the point of the maximum proportion of *lower* responses **(B)**; magnitude of effect is the difference between maximum and minimum of the response function; in this example, the SHPC is approximately at C# and the magnitude of effect is about 40%.

Proximity is not a valid cue for Shepard tones forming a tritone interval (separated by 6 semitones; tritone pairs; see Figure 1B). Accordingly, pitch judgments of tritone pairs should be ambiguous. However, Deutsch (1986, 1988) revealed that participants were able to judge the pitch direction of tritone pairs reliably, based on the pitch class of the first tone (tritone paradox). The tritone paradox is, at best, only moderately affected by the spectral structure of Shepard tones (Deutsch, 1986, 1987, 1988, 1991; Cohen et al., 1994; Giangrande, 1998; Repp and Thompson, 2010), ruling out a simple low-level mechanism. Interestingly, the resulting ascending-descending patterns differ from person to person, depending on the linguistic background (Deutsch et al., 1987; Deutsch, 1991; Dawe et al., 1998; Giangrande, 1998; Chalikia and Leinfelt, 2000; Chalikia et al., 2000, 2001). Figure 2 describes the extraction of the subjectively highest pitch class (SHPC) and the magnitude of effect from these patterns. The SHPC is given by the direction of the resultant vector of the data points (for more details see Fisher, 1993; Repp and Thompson, 2010). The magnitude of effect is the difference between maximum and minimum of the response pattern.

Another interesting finding is that tritone pairs are influenced by prior context (Repp, 1997; Giangrande et al., 2003; Englitz et al., 2013; Chambers and Pressnitzer, 2014; Chambers et al., 2017). Repp (1997) found that tritone pairs were affected by a prior Shepard tone. Recently, studies have shown that preceding tone sequences also caused adaptation (Dawe et al., 1998; Malek and Sperschneider, 2018) and other context effects in tritone pairs (Englitz et al., 2013; Chambers and Pressnitzer, 2014; Chambers et al., 2017). Two computational models were recently published to explain these context effects (Huang et al., 2015; Chambers et al., 2017).

This study intends to contribute to the theoretical discussion about the origin of the tritone paradox. One explanation for the tritone paradox posits that pitch judgments are based on the comparison of the pitch classes with an internal pitch class template that reflects an abstract form of an implicit absolute pitch and is possibly acquired through language experience (Deutsch, 1991; Deutsch et al., 2004). In other words, participants are assumed to compare two pitch classes instead of two pitch heights, which is usually assumed for unambiguous *normal* tones (e.g., musical tones), suggesting the importance of pitch class instead of pitch height. Thus, such a pitch class comparison mechanism differs from the known comparison mechanism in ordinary harmonic tones and represents a highly specific mechanism that is proposed for the selected class of Shepard tone comparisons. A contrasting explanation for the tritone paradox postulates no specific mechanism for Shepard tones but explains it with common pitch-processing theories (Terhardt, 1991; Cohen et al., 1995). Terhardt (1991), in particular, explained the tritone paradox with his *virtual pitch theory* (VPT; Terhardt et al., 1982a,b), postulating that listeners extract fundamental frequencies from Shepard tones to determine their pitch heights. Here, I propose a model for the tritone paradox that emphasizes such a psychoacoustic explanation.

There is evidence that listeners can extract fundamental frequencies from Shepard tones. Terhardt et al. (1986) and Repp and Thompson (2010) conducted a pitch-matching task (listeners had to match frequencies of pure tones or harmonic complex tones to Shepard tones). Matched frequencies corresponded to Shepard tone components in different octaves. They ranged from 200 to 1500 Hz and concentrated around 300 Hz. Matched frequencies were in accordance with the predictions of the Terhardt pitch-processing model (Terhardt et al., 1982b). The current paper intends to replicate the finding that frequency matches for single Shepard tones are ambiguous with respect to octave position and, in the next step, to show that this ambivalence in octave position can cause the tritone paradox.

Repp and Thompson (2010) investigated whether the results of the pitch-matching task predict the results of the standard tritone paradox paradigm. Participants were asked for the best and the second best match out of four possible harmonic comparison tones (in different octaves) for each of the 12 Shepard tones. The authors estimated the subjective pitch height of each Shepard tone by calculating the sum of the MIDI pitch number of the matches, weighted by participants' confidence rating, and they compared it with the subjective pitch height measured with the standard tritone paradigm. Considering the averaged sample data, both measures were consistent, indicating that the ambiguity found in matching tasks causes the ambiguity in the tritone paradox. However, there was no consistency in the individual data, indicating that the typical phenomenon of the tritone paradox might not be assessable with matching tasks. Thus, much uncertainty still exists about the relationship between the ambiguity in matching tasks and the tritone paradox.

To conclude, the studies supporting the psychoacoustic explanation of the tritone paradox have focused on the pitch of single Shepard tones. However, no study up to now has shown that the psychoacoustic approach can explain the typical response patterns of the standard tritone paradox paradigm (tone-pair comparison task). This paper focuses on a psychoacoustic explanation for the pair-comparison patterns. As stated above, the pitch-matching experiments of Terhardt et al. (1986) and Repp and Thompson (2010) have revealed that Shepard tones are perceived as harmonic complex tones but are more ambiguous. This paper intends to explain the tritone paradox by considering Shepard tones as harmonic complex tones. I introduce an algorithm, the threshold model, that assumes that Shepard tones are processed like harmonic complex tones and provides predictions for the typical tritone paradox response patterns. The algorithm is based on the comparison of probabilistic fundamental frequency estimates. It combines psychoacoustic and physiological findings and concepts with the classical threshold theory (Gescheider, 1997) and probability theory. The aim is not to develop a new general elaborate pitch-processing theory; however, the main assumptions of the threshold model should not contradict the main ideas of Terhardt's algorithm or other pitch-processing theories. In the literature, there exist two main types of pitch-processing theories (for an overview, see Cheveigné, 2005, 2010): pattern-matching theories (Schroeder, 1968; Goldstein, 1973; Wightman, 1973; Terhardt, 1974) and autocorrelation theories (Licklider, 1951; Meddis and Hewitt, 1991; Meddis and O'Mard, 1997). The threshold model belongs to the pattern-matching type, referring to the VPT (Terhardt et al., 1982a). However, it is greatly simplified, especially in the pattern-matching processing stage, where the harmonic template is simply realized by taking the lowest frequency component and is restricted to harmonic complex tones with resolved components. The aim of the paper is to show that even such a simple mathematical model based on basic pitch-processing mechanisms can explain the main findings of the tritone paradox.

### 1.1. Threshold model

**Figure 3:**
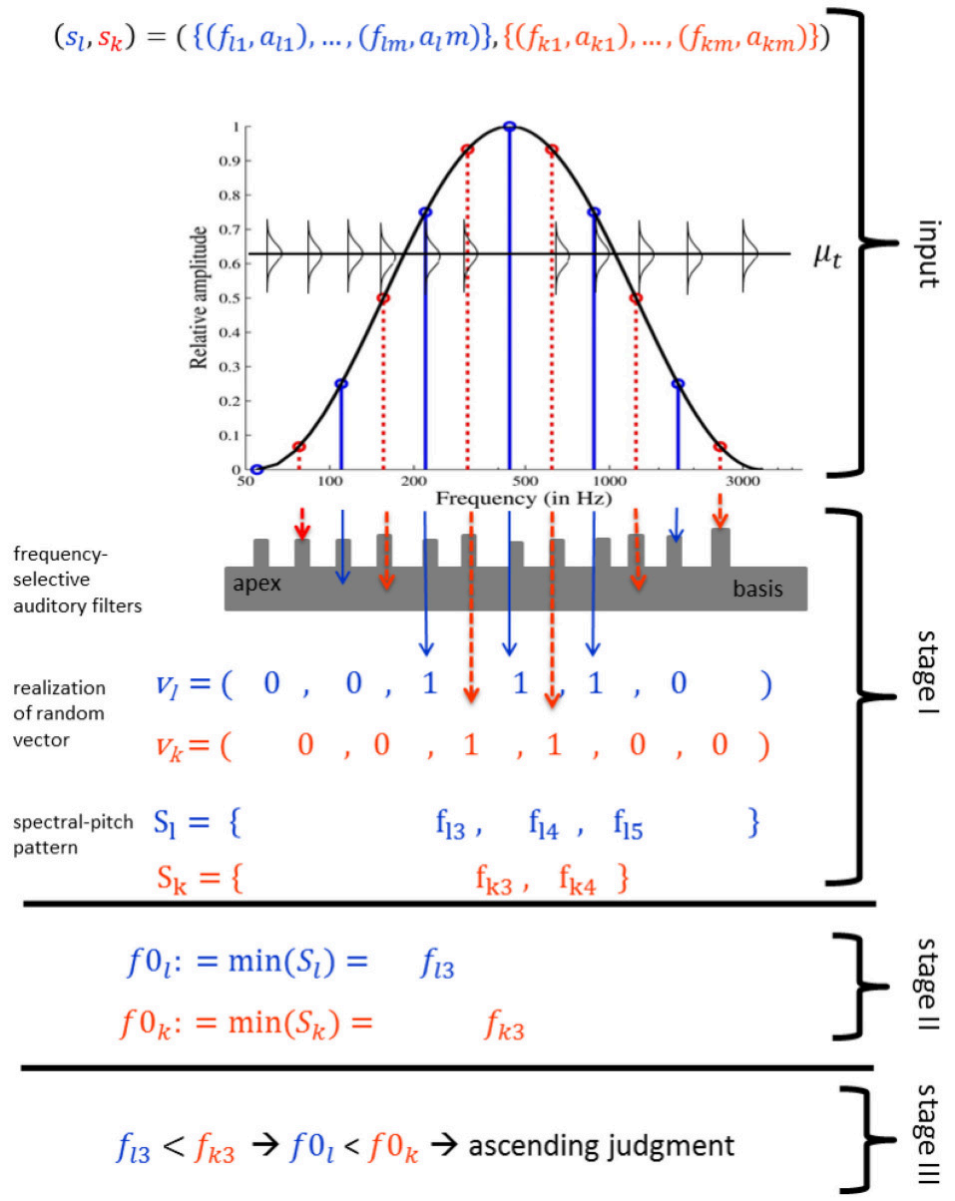
Threshold model algorithm for a Shepard tone pair (*s*_*l*_, *s*_*k*_): three components of the first Shepard tone (blue solid line) and four components of the second Shepard tone (red dotted line) are filtered in the first processing stage, resulting in three unfiltered first tone components and two unfiltered second tone components. The frequency of the lowest unfiltered component is assigned the role of fundamental frequency in stage II. Because the first tone fundamental frequency is lower than the second tone fundamental frequency, the model output is an ascending judgment (stage III).

Figure 3 provides an overview of the threshold model algorithm (see Appendix for details and reasoning). The threshold model estimates the Shepard tone pitch heights by a probabilistic fundamental frequency extraction mechanism. Commonly, harmonic complex tones consist of a fundamental frequency, associated with pitch height and several harmonics, which are integer multiples of the fundamental frequency and are associated with timbre. The fundamental frequency is often, but not necessarily, the lowest tone partial. Even when the fundamental frequency is deleted or masked, the pitch height corresponds to the fundamental frequency (missing fundamental, Shouten, 1940; Licklider, 1959), resulting in the relatively robust pitch height found for harmonic complex tones. The threshold model supposes that Shepard tones' fundamental frequencies determine their pitch heights. Pitches of harmonic complex tones are relatively robust, because the fundamental frequencies can be reconstructed from the harmonics, even when the tones fail to comprise the fundamental frequency, which is the greatest common divisor of the harmonic frequencies. The greatest common divisor of Shepard tone components is the frequency of the lowest component due to the octave-spaced frequency components. In contrast to harmonic complex tones, filtering out the lowest frequency component changes the fundamental frequency and, therefore, Shepard tones' pitch height. Thus, the lowest frequency component determines Shepard tones' pitch height.

**Figure 4:**
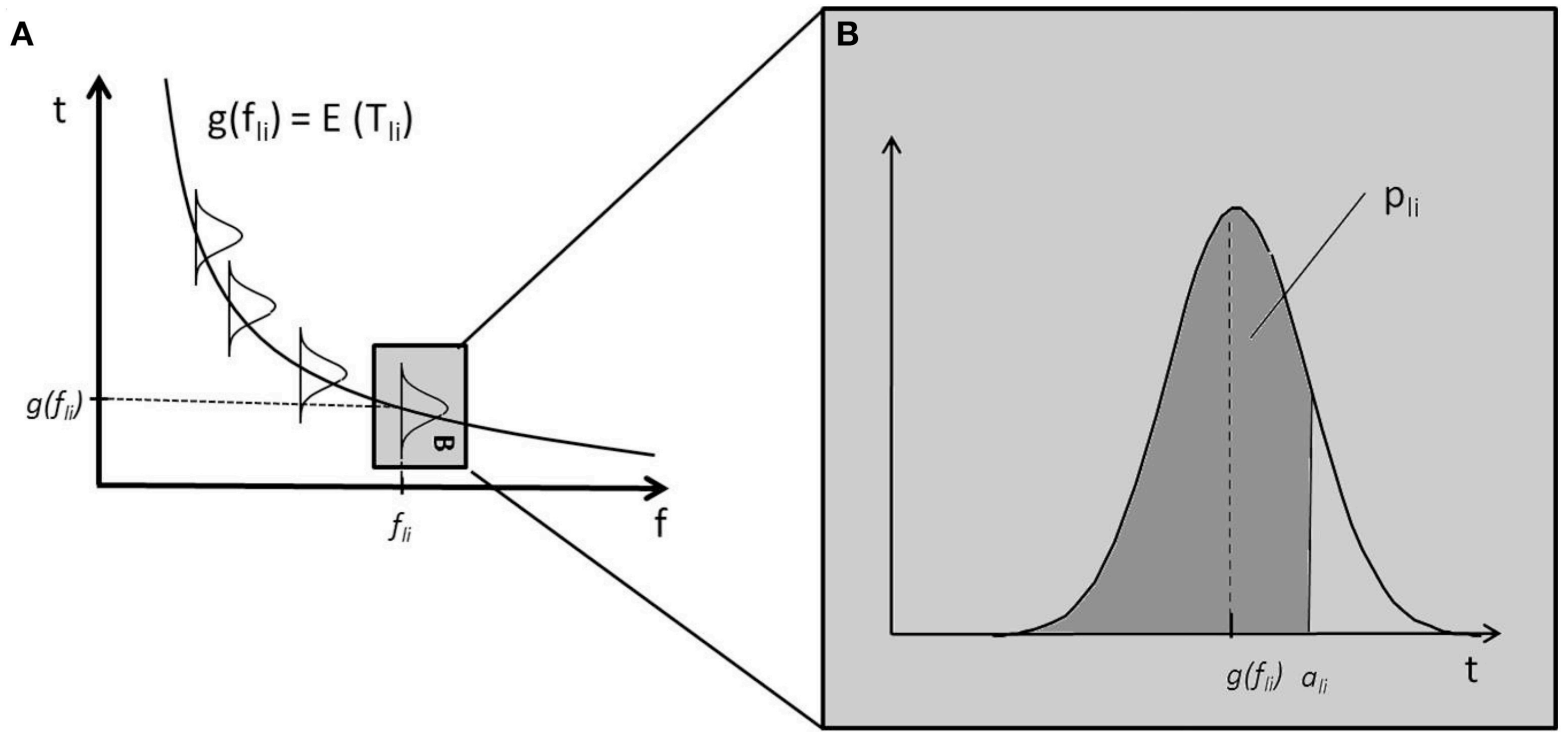
Example of a monotonically decreasing threshold function *g* and sketched distributions of threshold values **(A)**; the distribution of *T*_*li*_ corresponding to the component *c*_*li*_ has an expected value μ_*t*_ = *g*(*f*_*li*_) gray box in **(A)**, enlarged in **(B)**; the probability that the component *c*_*li*_ is not filtered, *p*_*li*_, is the area under the probability density function of *T*_*li*_ for *t* ≤ *a*_*li*_ (the dark gray area in **B**).

The fixed bell-shaped envelope attenuates low (and high) components of Shepard tones, resulting in uncertainty about which component is the lowest audible (and, hence, relevant) component. The threshold model implements a probabilistic component filtering mechanism. Frequency components with frequency *f* are filtered out when their amplitudes are beneath specific threshold values, which are realizations of random variables *T*. Their expected values μ_*t*_ are determined by a so-called threshold function *g*. Figure 4 shows a frequency-dependent threshold function *g*(*f*). It also shows that the probability that a component is not filtered out corresponds to the area under the probability density function. The probability that a Shepard tone has a specific pitch height is given by the probability that the corresponding frequency component is the lowest nonfiltered component. The probability of an ascending judgment is the probability that the pitch height of the first Shepard tone is lower than the pitch height of the second Shepard tone.

### 1.2. Threshold function g

Within the threshold model, it is assumed that individual differences in the tritone paradox are caused by individual differences in the threshold function. The threshold function can be considered as an implementation of Terhardt's internal spectral weighting function, which causes a specific frequency region to be particularly sensitive (preference region). The mathematical description is given in the Appendix.

## 2. Pretest: pitch-matching experiment

An initial pitch-matching experiment was conducted to replicate the findings of Repp and Thompson (2010) and Terhardt et al. (1986) that frequency matches correspond to Shepard tones' frequency components and are around 300 Hz. Furthermore, the matched frequencies were compared to the predictions of Terhardt's VPT, which provides so-called spectral pitches (SPs) and virtual pitches (VPs). Spectral pitches corresponded directly to partials and were weighted by a so-called internal spectral weighting function. Virtual pitches were determined by a pattern-matching procedure, a subharmonic coincidence detection. Here, all subharmonics of salient SPs are VP candidates, which are weighted. Only the most salient VPs are of interest. The VPT provides a weighted spectral pitch and a weighted virtual pitch pattern, which are both relevant for pitch. Terhardt (1991) postulated that Shepard tones' pitches are mostly determined by their VPs and not by their SPs. Thus, it is assumed that pure tone matching corresponds to the VPs rather than to the SPs.

### 2.1. Methods

#### 2.1.1. Participants

Normal-hearing, undergraduate students from the Martin Luther University Halle-Wittenberg (*n* = 8; 7 women) participated in the study. They were aged between 19 and 36 years (*M* = 24.28, *SD* = 5.76). No professional musician participated in the study. At the time of the survey, four participants had never played an instrument; two had learnt an instrument but did not play regularly anymore; two played an instrument regularly. All participants lived and were raised in Germany. They received credit points for their psychology courses in exchange for their participation, as is approved by the study board of the Department of Psychology, Martin Luther University Halle-Wittenberg. The experiments conducted do not require formal ethical approval according to the German law and institutional requirements. Before participation, the students were informed that the collected data would be used in an anonymous form for publication. All students participated voluntarily and were free to opt out with no negative consequences at any time of the experiment. The study was conducted in accordance with the declaration of Helsinki and the University Research Ethics Standards.

#### 2.1.2. Equipment

The experiment was run on an Intel Core 2 Duo Windows computer containing a VIA high definition audio sound card. Participants listened to a stereo signal via Sennheiser HD 202 earphones (Frequency response, -8.96, +3.21 dB in 100–10 kHz Center/summary HDM1) in an acoustically silenced room at the University. The experiment was presented by Pxlab (Irtel, 2007). The stimuli were synthesized in Matlab (Version 7.40.287).

#### 2.1.3. Stimuli

The Shepard tones were constructed according to the specifications of Deutsch et al. (1987). Each Shepard tone corresponded to one of the 12 chromatic pitch classes (*C*, *C#*, …, *B*) and consisted of six sinusoidal octave-spaced components (see Figure 1). The frequency *f*_*li*_ of the i^th^ component of the Shepard tone *l* for all *i* = 1, …, *m*, *l* = 1, …, *l*_*max*_ is

(1) $$\begin{array}{r} f_{li}:=f_{l1}\cdot 2^{i-1}, \end{array}$$

where

(2) $$\begin{array}{r} f_{l1}:=f_{\text{min}}\cdot 2^{\left(l-1\right)/l_{\text{max}}}. \end{array}$$

Their amplitudes were determined by a fixed, bell-shaped spectral envelope. The general form of this envelope is described by the following equation:

(3) $$\begin{array}{r} A\left(f_{i}\right)=0.5-0.05\cdot cos\left(\frac{2\pi }{\gamma }\cdot log_{\beta }\left(\frac{f_{i}}{f_{\text{min}}}\right)\right), \end{array}$$

where A(f) is the relative amplitude of a sinusoid at frequency f in Hz, β is the frequency ratio formed by adjacent sinusoids (β = 2, hence octave spacing), γ is the number of β cycles spanned (γ = 6), and *f*_min_ is the minimum frequency for which the amplitude is nonzero (*f*_min_ = 32.70Hz, generating an envelope centered at *C*_4_: 262 Hz).

Six Shepard tones were used. Their lowest frequencies were 34.65 Hz (C#), 38.89 Hz (D#), 43.65 Hz (F), 48.00 Hz (G), 55.00 Hz (A), and 61.74 Hz (B), respectively. All tones were 800 ms in duration with 53 ms sinusoidal amplitude ramps at the beginning and at the end. The sample rate was 44.1 kHz and the sound level was about 55 dB.

#### 2.1.4. Research design and procedure

On each trial, a Shepard tone and a sinusoidal comparison tone were presented, separated by a silent period of 200 ms. Participants were asked to judge whether the second tone was *higher* or *lower* in pitch than the first tone by pressing the up arrow and down arrow on the number pad. They received visual feedback about the key which they had pressed but no feedback about the correctness of their answer. Participants were able to repeat the tone pairs as often as they wished.

The frequency of the comparison tone was determined in each trial using an up-down staircase method (Levitt, 1971) implemented in PxLab (Irtel, 2007). Each adaptive sequence started with a randomly chosen frequency (310–910 Hz). When the participant answered that the comparison tone was *higher* or *lower* than the Shepard tone, the frequency was reduced or increased by its *stepsize T*, respectively. This stepsize was reduced at *turnpoints*, trials in which the response direction changed (from *higher* to *lower* responses or *vice versa*). Each adaptive sequence started with an initial step size *T*_0_ of 200 Hz. Subsequently, the stepsize *T*_*t*_ in trial *t* of the adaptive sequence was computed as $T_{t}=\frac{T_{0}}{t}$. The adaptive sequence stopped when four turnpoints occurred at a step size *T*_*t*_ smaller than 20 Hz or after forty trials. The frequency of the comparison tone could vary in the range of 50–3,000 Hz. The arithmetic mean and the standard deviation of the four last turning points in an adaptive sequence were used to estimate the PSE and its standard error (Wetherill, 1963).

There were four adaptive sequences for each Shepard tone (C#, D#, F, G, A, and B). In two adaptive sequences, the Shepard tone followed the comparison tone and in the other two, the comparison tone followed the Shepard tone to control for order effects. Data for each participant were collected on each of the two different day sessions (1 h per session; 900 trials per session). Participants received a short training with the task at the beginning of the first session.

### 2.2. Results

**Figure 5:**
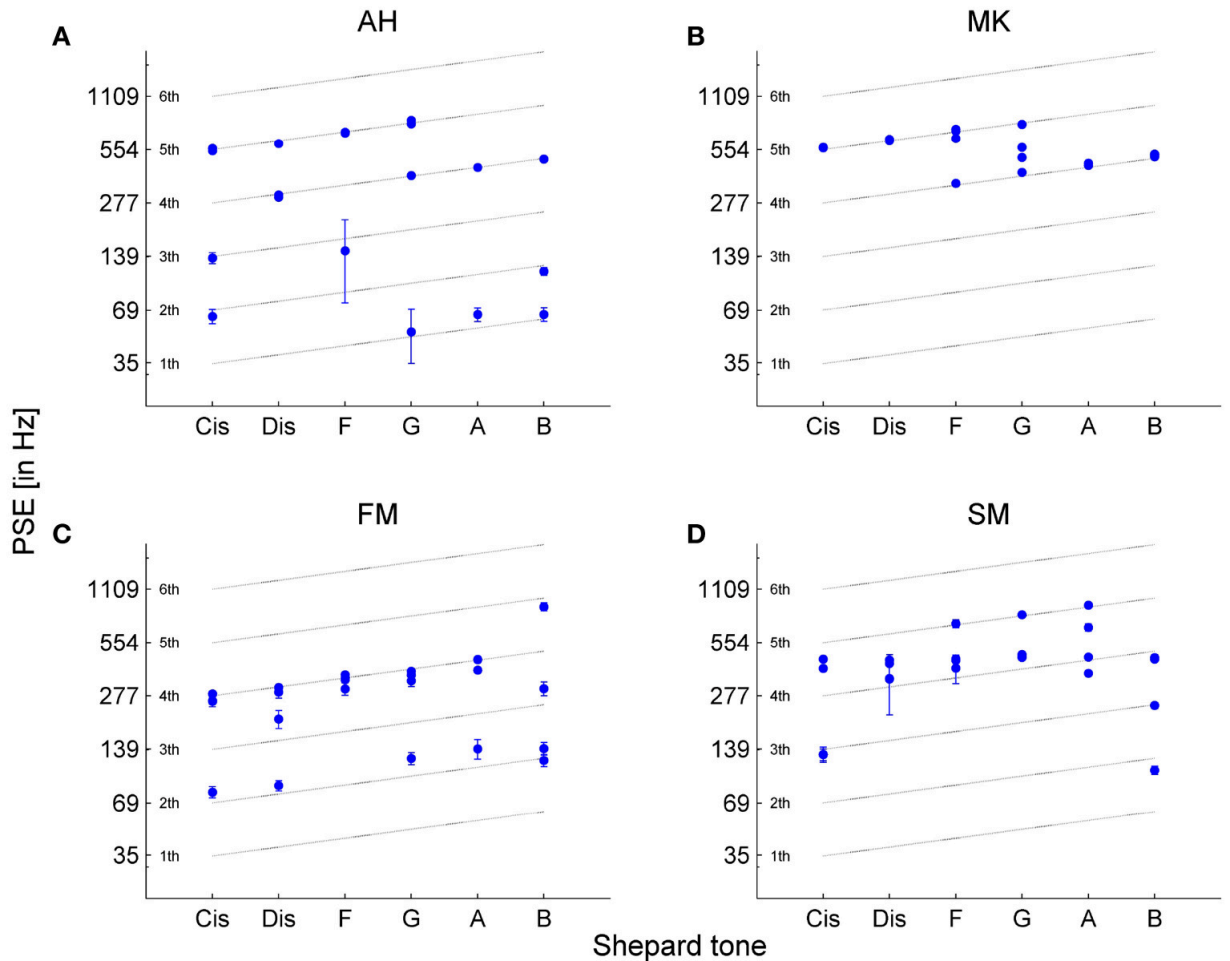
Point of subjective equality (PSE) estimates of the four adaptive sequences for each Shepard tone for **(A)** participant *AH*, **(B)** *MK*, **(C)** *FM* and **(D)** *SM*. The PSEs were estimated by the mean of the last four turnpoints in the up-down staircase method. The error bars represent the standard error of the four turnpoints used for PSE estimation. In some cases, standard errors are too small to be visible. There are less than four data points in some cases because of several reasons: the turnpoints were not enough to estimate the PSE, and similar PSE estimates result in overlaid data points.

The estimated PSEs corresponded to Shepard tones' frequency components in different octaves, revealing the octave ambiguity in pitch height of single Shepard tones (see Figure 5). In some cases, it was impossible to estimate PSEs because of missing turnpoints (Figure 5A). In other cases, the standard errors of the PSE estimates were large, indicating that the frequencies adjusted by the up-down staircase method were not converging over the trials (Figures 5A,D). The PSEs were estimated between 52 Hz and 1356.67 Hz. Most PSEs were between 200-600 Hz, with a preference for the fourth component (around 300 Hz), corresponding to the envelope centered at 264 Hz (*C*_4_). Interestingly, preferred matches at 300 Hz were also found by Terhardt et al. (1986) and by Repp and Thompson (2010).

**Figure 6:**
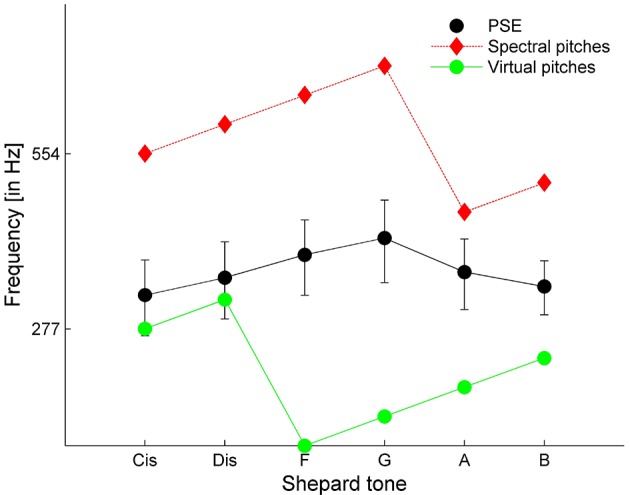
Averaged PSE estimates compared to the spectral and virtual pitches with the highest weight predicted by the VPT. Error bars represent the standard error (*n* = 8).

Comparing the averaged PSEs with the prediction of the VPT (predicted by Terhardt, 1990) revealed that the PSEs were between the SPs and VPs. The absolute values were more similar to the VPs than to the SPs (see Figure 6). However, the highest PSE was at Shepard tone *G*, which was in accordance with the highest SP and not the highest VP (*D#*).

### 2.3. Discussion

The experiment showed that the listeners match frequencies of pure tones to octave components in different octaves, indicating octave ambiguity in pitch height for single Shepard tones. However, it remains unclear whether this ambiguity is a genuine property of Shepard tones or is only an artifact of the matching task. For some participants, the up-down staircase method failed to succeed in converging on a frequency. Furthermore, the PSE estimates varied significantly for some participants, indicating a possible difficulty in comparing sounds of different timbre, which typically results in increased errors, especially, for nonmusicians (Seither-Preisler et al., 2007).

A further problem of the method is whether the frequency matches correspond to the “true” pitch or to frequency components emphasized by the sinusoidal comparison tone; that is, the problem is whether the pitch height assessable in the pure tone matching task corresponds to that in the tone comparison task. Terhardt (1991) postulated that Shepard tone pitches are mostly determined by their VPs and not by their SPs. The experiment showed that, overall, the frequency matches failed to correspond to VPs, indicating that at least some matches correspond to SPs, possibly emphasized by the frequency of the comparison tone. Possibly, participants were distracted by SPs, which increased noise and caused the ambiguity in octave position. To conclude, it remains unclear whether the octave ambiguity found in single Shepard tones is only an artifact of the pure tone matching method.

## 3. Tone-pair comparison task

The goal of the second experiment was to ascertain whether the ambiguity found in the pitch height of single Shepard tones accounts for the tritone paradox, providing support for the psychoacoustic account. First, the highest Shepard tone assessable in the pure tone matching task should correspond to that in the tone-pair comparison task. Thus, the highest Shepard tone should be around *G* for a stimulus set generated under a fixed envelope centered at 261 Hz. Second, the threshold model, implementing pitch ambiguity in single Shepard tones, should predict the typical response patterns of the tritone paradox.

The typical finding when testing tritone pairs has been that some tone pairs are clearly judged as rising in pitch, some are ambiguous (i.e., in some cases judged as rising and in the other as falling) and some are clearly judged as falling in pitch, resulting in a sigmoid response function (Deutsch, 1991; Repp, 1997). Although the sigmoid form is a typical finding of the tritone paradox, most theories have not considered it. For example, Terhardt (1991) considered the dominant virtual pitches, causing a staircase response pattern but not the sigmoid pattern. Thus, the threshold model ought to predict the typical sigmoid function.

A much debated question is whether the spectral envelope affects the tritone paradox. Shepard tones were constructed under a fixed spectral envelope. Shifting the envelope center on the frequency axis changes the amplitude relation of the Shepard tones' components (see Figure 1). In most studies, the pitch judgments of most participants were, at best, only minimally affected by envelope shifts (Deutsch, 1986, 1987, 1988, 1991; Cohen et al., 1994; Giangrande, 1998; Repp and Thompson, 2010). Some studies, however, found pronounced envelope effects for at least some participants (Repp, 1994, 1997; Krüger and Lukas, 2002; Krüger, 2011). Repp (1997) quantified envelope effects by averaging *lower* response rates across envelope sets not as a function of the pitch classes but as a function of the clockwise distance of these pitch classes from the envelope center (e.g., the Shepard tone *C* is shifted by 0 semitones whereas the Shepard tone *C#* is shifted by 1 semitone from envelope center *C*_4_). In particular, Repp (1997) revealed that the individual highest pitch classes were shifted by about 6 semitones from the envelope center. The threshold model should predict that the response pattern depends on the pitch class and on the envelope center. Empirical evaluation and simulations were conducted in order to determine whether this is dependent on the form of the threshold function.

### 3.1. Methods

#### 3.1.1. Participants

Normal-hearing, undergraduate students from the Martin Luther University Halle-Wittenberg (*n* = 29; 22 women) participated in the study. They were aged between 18 and 31 years (*M* = 21.7, *SD* = 3.09). No professional musician participated in the study. At the time of the survey, most participants had some musical experience. Only four participants had never played an instrument or sung regularly. The participants played an instrument on average 1.57 h a week (*SD* = 2.53) or sung 0.69 h a week (*SD* = 1.32) in a choir or received singing lessons. On average, listeners made 5.60% errors (*SD* = 7.15%, range: 0−25%) in a pure tone discrimination pretest. All participants lived and were raised in Germany. Payment and ethical standards were the same as in the first experiment.

#### 3.1.2. Stimuli

The Shepard tones were constructed in the same way as in the pitch-matching experiment (see Equations 1–3). The following 12 tritone pairs were formed: *C*−*F#*, *C#*−*G*, *D*−*G#*, *D#*−*A*, *E*−*A#*, *F*−*B*, *F#*−*C*, *G*−*C#*, *G#*−*D*, *A*−*D#*, *A#*−*E*, and *B*−*F*. Each one of the tritone pair was synthesized under six spectral envelopes centered at different envelope centers on the frequency axis. The envelope centers were chosen to cover a wide frequency region (see Figure 12) to test different threshold function forms. There was one envelope in the low frequency region centered at 65.41 Hz (*C*_2_, *f*_*min*_ = 8.18), four envelopes in the middle frequency region centered at 261 Hz (*C*_4_, *f*_*min*_ = 32.70), 370 Hz (*F#*_4_, *f*_*min*_ = 46.25), 440 Hz (*A*_4_, *f*_*min*_ = 55), and 523.25 Hz (*C*_5_, *f*_*min*_ = 65.41), and one envelope in the high frequency region centered at 1244.51 Hz (*D#*_6_, *f*_*min*_ = 155.56). Each Shepard tone (sampling rate = 44.1 kHz) lasted 800 ms and had a constant amplitude, with the exception of 53.6 ms sinusoidal ramps at onset and offset to prevent clicks. Subsequent Shepard tones were separated by a 200 ms interstimulus interval. They were presented at a volume of approximately 55 dB sound pressure level (SPL).

White noise (20–10,000 Hz; level = 40 dB SPL) was presented in the background of each tritone pair (Zwicker and Feldtkeller, 1967; Hartmann, 1998). Sample audio files are provided in the Supplementary Material. Background noise masks the potential products of nonlinear distortions in the ear canal such as combination tones (Zwicker and Feldtkeller, 1967; Hartmann, 1998) and attenuates the frequency dependence and interindividual differences of single-frequency hearing thresholds (Zwicker and Feldtkeller, 1967), facilitating the fitting of the threshold function.

#### 3.1.3. Pure tone pretest

To measure the ability to discriminate pitch direction for non-ambiguous stimuli, the experiment started with a pure tone pretest, comprising 16 randomized different tone pairs, lasting 800 ms with a level of 55 dB SPL. On each trial, a standard and a comparison tone were presented, separated by a 200 ms silent interval. Participants had to judge whether the comparison tone was *higher* or *lower* in pitch than the standard tone. They could repeat the tone pair as often as they wished by pressing a repeat key. The standard tone always preceded the comparison tone. The standard tones' frequencies corresponded to the fourth component of four randomly chosen Shepard tones, because the pitch-matching experiment suggested that Shepard tones' pitches lie in this region. There were four standard tones with frequencies of 261.62 Hz, 277.18, 392, and 493.88 Hz, which corresponded to the fourth component of the Shepard tones *C*, *C#*, *G*, *B*. The frequency of the comparison tones ranged from 207 Hz (*G#*_3_) to 622 Hz (*D#*_5_). The frequency differences between standard and comparison tone ranged from 1–4 semitones. The standard tones with frequencies 261.62 and 392 Hz formed decreasing tone pairs; the standard tones with frequencies 277.18 Hz and 493.88 Hz formed increasing tone pairs. White noise was presented in the background of each tone pair. No tone pair was presented twice, except when participants pressed the repeat key. Thus, the pretest consisted of at least 16 trials.

#### 3.1.4. Equipment

The equipment was the same as that used in the pure tone matching experiment.

#### 3.1.5. Design and procedure

One tritone pair was presented in each trial. After the presentation, the participants were asked to judge whether the second tone was *higher* or *lower* in pitch than the first tone. After response, the next trial started immediately.

The participants listened to 12 (pitch classes) × 6 (envelope centers) × 30 (repetitions) experimental trials (2160 trials) and 12 (pitch classes) × 6 (envelope centers) × 2 (repetitions) practice trials (144 trials). Thus, each tritone pair was presented 32 times, and the first two presentations of each tone pair were practice trials and were excluded from data analysis. The trials were presented in blocks according to the envelope center. The order of the blocks and the order of the tritone pairs within each block were randomized for each participant. Participants could rest after each block of trials and could continue with the experiment when they wanted. The experiment was divided into two sessions, each lasting 1.5 h and separated by at least 1 day.

### 3.2. Results

Categorical lower/higher responses were analyzed with logistic regression (Jaeger, 2008).

#### 3.2.1. Data analysis

##### 3.2.1.1. Sample data.

Averaged *lower* responses as a function of the first Shepard tone were sigmoid and less pronounced for all envelope centers (see Figure 8), indicating high variability within or between the listeners. The highest Shepard tones depended on the envelope center (see Table 1). The highest Shepard tone for the *C*_4_-envelope (at 261 Hz) was at 7.43 (*F#*−*G*), corresponding, approximately, to the highest Shepard tone found in the pure tone matching experiment.

**Table 1:** Highest Shepard tones in the data predicted by the threshold model using a horizontal and a logistic threshold function for the six envelope centers.

**Envelope center**	**Data**	**Horizontal**	**Logistic**
*C*_2_	3.68 (D-D#)	5.73 (E-F)	3.68 (D-D#)
*C*_4_	7.43 (F#-G)	5.73 (E-F)	6.54 (F-F#)
*F#*_4_	11.78 (A#-B)	11.73 (A#-B)	12.54 (B-C)
*A*_4_	4.12 (D#)	2.73 (C#-D)	3.54 (D-D#)
*C*_5_	5.74 (E-F)	5.73 (E-F)	6.54 (F-F#)
*D#*_6_	1.79 (C-C#)	8.73 (G-G#)	9.54 (G#-A)

*The horizontal threshold function parameters were ${\hat{\mu }}_{t}=0.58$, ${\hat{\sigma }}^{2}=0.19$ and the logistic threshold function parameters were â = 5.67, $\hat{b}=0.13$, $\hat{q}=0.62$, ${\hat{\sigma }}^{2}=0.18$*.

A multilevel (i.e., mixed-effects) logistic regression model (Jaeger, 2008) was fitted, with initial pitch class (*C*, *C#*, *D*, *D#*, *E*, *F*, *F#*, *G*, *G#*, *A*, *A#*, *B*) and envelope center (*C*_2_, *C*_4_, *F#*_4_, *A*_4_, *C*_5_, *D#*_6_) as predictors and averaged lower responses as the outcome. The pitch classes significantly affected the proportion of *lower* responses, χ(1)^2^ = 75.96, *p* < 0.0001. Pitch class effects were quantified by averaging *lower* response rates across the envelope sets as a function of pitch classes. The difference of the maximum and the minimum of this response function was taken as the magnitude of pitch class effect. For continuously varying measures of the highest pitch class, the rotation angle of the resultant vector was calculated from the averaged *lower* response rates as a function of Shepard tones' pitch classes (see Figure 2, Fisher, 1993; Repp and Thompson, 2010). This measure ranged between 1 and 12 and corresponded to the consecutive pitch classes, for example 1 corresponded to *C*, 2 to *C#*, etc. The highest pitch class was 3.52 (between *D* and *D#*), and the magnitude of effect was small (10.15%). The envelope centers also significantly influenced the proportion of *lower* responses, χ(1)^2^ = 21.31, *p* = < 0.0001. There were less *lower* responses under the envelope centered at *C*_2_ (*M* = 47.71%) than under those in the middle and high frequency regions (*C*_4_; *M* = 50.22%, *F#*_4_: *M* = 51.78%, *A*_4_: *M* = 51.23%, *C*_5_: *M* = 50.91%, *D#*_6_: *M* = 51.16%). The interaction between pitch classes and envelope centers was also significant, indicating that the highest pitch class depended on the envelope center (envelope effects), χ(1)^2^ = 51.38, *p* = < 0.0001. As described above, envelope effects were quantified using the procedure described by Repp (1997). The highest pitch class was 4.72 semitones shifted from the envelope center, and the magnitude of effect was slightly larger than the magnitude of pitch class effect (16.44%).

##### 3.2.1.2. Individual data

**Figure 7:**
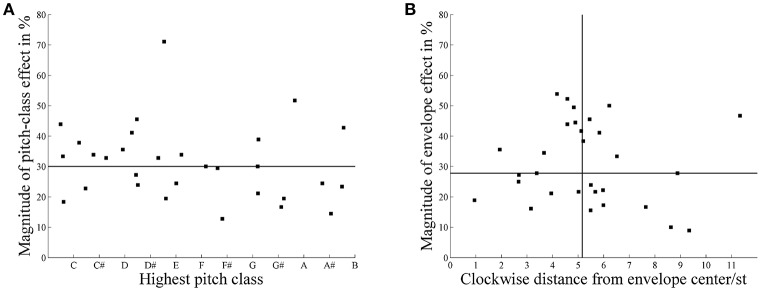
**(A)** Distribution of individual highest pitch classes and the magnitude of pitch class effect (*n* = 29). **(B)** Distribution of individual highest pitch classes shifted from the envelope center (in semitones, st) and the magnitude of envelope effects (*n* = 29). The horizontal line represents the median of the individual magnitude of effect. The vertical line represents the median of the clockwise distance by which the highest pitch class is shifted from the envelope center.

Pitch class and envelope effects were calculated individually for each participant to investigate whether the small effects were due to small individual effects or high individual variability. Considering pitch class effects, the individual highest pitch classes were distributed nearly uniformly across the whole pitch class circle (see Figure 7A); the Rayleigh test for non-uniformity of circular data (Berens, 2009) revealed that the distribution of highest pitch classes around the pitch class circle failed to significantly deviate from a uniform distribution, *R* = 0.36, *p* = 0.70, *n* = 29. Thus, the listeners failed to agree on which pitch class was the highest, despite having the same linguistic background.

Considering envelope effects, the individual highest pitch classes were shifted, on average, 5.20 semitones from envelope center (see Figure 7B); the Rayleigh test for non-uniformity of circular data revealed a significant deviation from a uniform distribution, *R* = 8.44, *p* < 0.0001, *n* = 29. Thus, the listeners agreed that the highest pitch class was shifted about 5 semitones from envelope center.

There was no significant correlation between pitch class and envelope effects, *r* = 0.24, *p* = 0.203 or between error rates of the pure tone discrimination test and the magnitudes of pitch class effect, *r* = 0.16, *p* = 0.405 or envelope effects, *r* = −0.292, *p* = 0.124.

#### 3.2.2. Model testing

The root mean squared deviation (*RMSD*) is used to validate a model's goodness of fit. This measure uses root mean squared error (*RMSE*) and, additionally, takes into account the number of model parameters *k* (Pitt et al., 2002): $RMSD=\sqrt{\left(SSE/\text{N-k}\right)}$.

##### 3.2.2.1. Sample data

**Figure 8:**
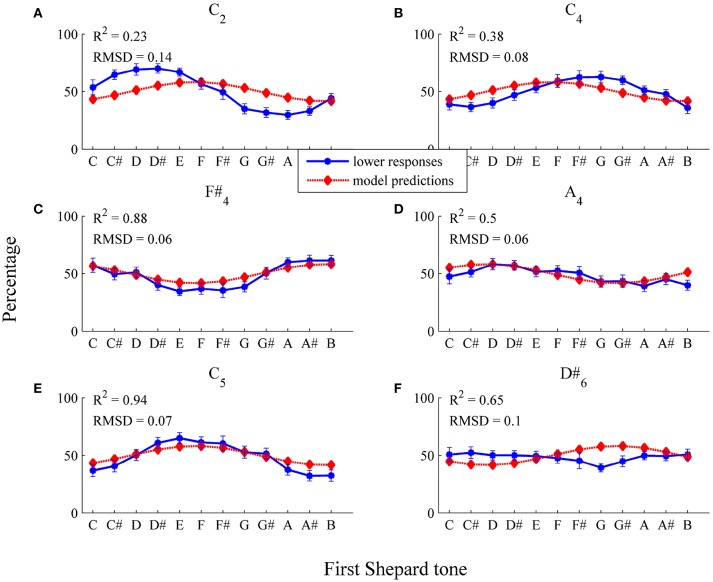
Fitting the horizontal threshold function: average percentages of *lower* responses (blue dots and unbroken line) in comparison to model predictions (red diamonds and dotted line; estimated model parameters: ${\hat{\mu }}_{t}=0.58$, ${\hat{\sigma }}^{2}=0.19$) for the six envelopes **(A)** *C*_2_, **(B)** *C*_4_, **(C)** *F#*_4_, **(D)** *A*_4_, **(E)** *C*_5_, **(F)** *D#*_6_. The error bars represent the standard error of mean (*n* = 29).

For testing the threshold model, it was necessary to choose the form of the threshold function (and the number of free parameters). The simplest assumption is that the threshold function (applied to each Shepard component) does not depend on frequency (horizontal threshold function). Thus, a horizontal function with the two parameters μ_*t*_ and σ^2^ was fitted, estimated by the least-squares method on the basis of the 72 sample data points (12 Shepard tones × 6 Envelope centers, *n* = 29). Model input was the logarithmic frequencies and the relative amplitudes of experimental stimuli. The threshold model predicted the sigmoid form of the response patterns and their shifts for the different envelope centers (see Figure 8 and Table 1, RMSD = 0.08, *R*^2^ = 0.31).

As it can be seen in Table 1, the predicted highest Shepard tones deviated by 2.02 semitones from the empirical highest Shepard tones. The largest deviation was found for the high envelope (*D#*_6_). However, the response function was nearly flat (see Figure 8F), and, thus, no reasonable highest Shepard tone was assessabled.

The model fit was poor for the lower envelope centers *C*_2_ and *C*_4_ (see Figures 8A,B), possibly because a horizontal threshold function is not appropriate in low frequency regions. Normally, psychoacoustic parameters depend on frequency, for example the hearing threshold is higher in low frequency regions than in middle frequency regions (Zwicker and Fastl, 1999). Thus, the expected value μ_*t*_ was modeled using the logistic function

(4) $$\begin{array}{r} \mu_{t}=q+\left(1-q-l\right)\cdot \left(1-\frac{1}{1+e^{-\left(\log_{2}\left(f\right)-a\right)/b}}\right), \end{array}$$

with *l* = 0 to implement a frequency-dependent threshold function. The logistic function is often used to fit a measured psychometric function to psychoacoustic data, because it has suitable general properties. For example, it begins at 0 and increases to 1 following a sigmoidal function.

**Figure 9:**
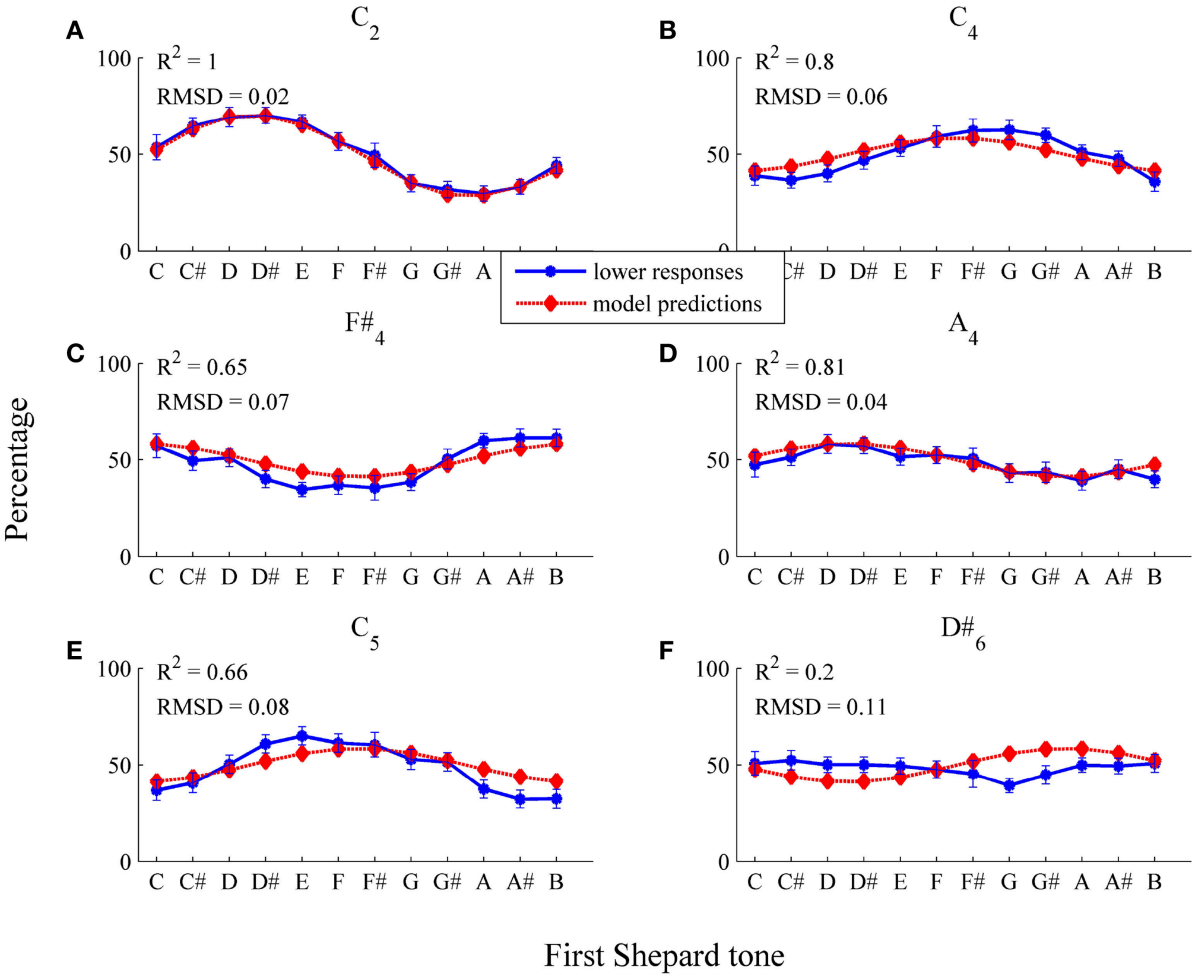
Fitting the logistic threshold function: average percentages of *lower* responses (blue dots and unbroken line) in comparison to model predictions for the six envelopes **(A)** *C*_2_, **(B)** *C*_4_, **(C)** *F#*_4_, **(D)** *A*_4_, **(E)** *C*_5_, **(F)** *D#*_6_ (red diamonds and dotted line; estimated model parameters: â = 5.67, $\hat{b}=0.13$, $\hat{q}=0.62$, ${\hat{\sigma }}^{2}=0.18$). The error bars represent the standard error of mean (*n* = 29).

Model predictions fitted well to data points in the middle and low frequency region (see Figure 9 and Table 1, RMSD = 0.06, *R*^2^ = 0.64). Particularly, model fit was improved for the *C*2 envelope (fit for the horizontal threshold function: *RMSD* = 0.14, *R*^2^ = 0.23; fit for the logistic threshold function: *RMSD* = 0.02, *R*^2^ = 1) and the *C*_4_ envelope (fit for the horizontal threshold function: *RMSD* = 0.08, *R*^2^ = 0.38; fit for the logistic threshold function: *RMSD* = 0.06, *R*^2^ = 0.8; also see Figures 9A,B), indicating that a falling, frequency-dependent threshold function is more appropriate than a horizontal, frequency-independent function for lower frequency regions. As can be seen in Table 1, the predicted highest Shepard tones deviated by no more than 1 semitone from the empirical highest Shepard tones, again, except for the *D#*_6_ envelope.

Comparing the estimated horizontal and logistic threshold functions shows differences in the low frequency region (see Figure 12). The logistic function depended on frequency only in the low frequency region. The estimated threshold functions were quite similar in the middle and high frequency regions. Components with relative amplitudes below 0.6 tend to be filtered out (horizontal: μ_*t*_ = 0.58; logistic: *q* = 0.62).

The estimated logistic threshold function was analyzed more closely (see Figure 12). The parameter *a*, which determined the position of the logistic function on the frequency axis, was estimated at 50.91 Hz (*a* = 5.67 was converted to the non-logarithmic frequency scale 2^5.67^ = 50.91), resulting in threshold function values nearly or even greater than one for frequencies lower than 50.91 Hz. Thus, components with frequencies lower than 50.91 Hz were probably filtered out, even if component amplitudes were maximal. Thus, the function parameter *a* can be considered as the lower limit of a preference region. The parameter *q*, which determined the lower limit of the range of the threshold function, was 0.62. The parameter *q* can be considered as a lower loudness boundary, because for components where the amplitude is smaller than *q*, the probability of not being filtered is rather small (< 0.5). The parameter *b*, which determined the slope of the threshold function, was 0.13, indicating a threshold function of moderate steepness.

##### 3.2.2.2. Individual data

**Figure 10:**
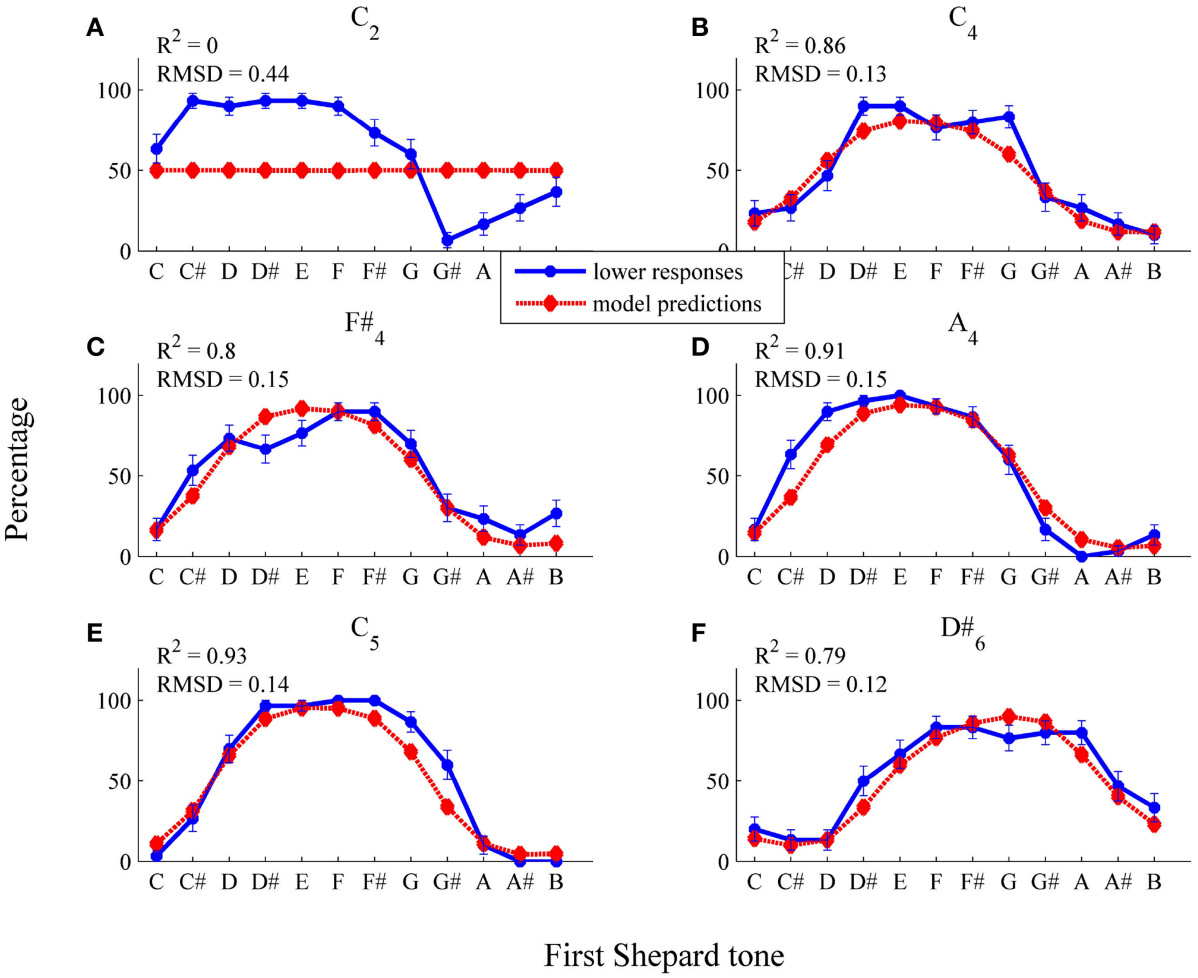
Fitted pronounced pitch class effects (participant *PG*): average percentages of *lower* responses (blue dots and unbroken line) in comparison to model predictions for the six envelopes **(A)** *C*_2_, **(B)** *C*_4_, **(C)** *F#*_4_, **(D)** *A*_4_, **(E)** *C*_5_, **(F)** *D#*_6_ (red diamonds and dotted line; estimated model parameters: â = 8.67, $\hat{b}=0.11$, $\hat{q}=0.29$, $\hat{l}=-0.57$, ${\hat{\sigma }}^{2}=0.27$).

To fit individual threshold functions, the four parameters of the logistic function (see Equation 4) and σ^2^ were estimated by the least-squares method on the basis of the 72 individual data points (12 Shepard tones × 6 envelopes, *n* = 30). As can be seen in Figure 10, the general trend of the responses of the participant PG (*R*^2^ = 0.79), who showed pronounced pitch class effects (71.11%), was predicted well by the threshold model. Except from the *C*_2_ envelope, the threshold model predicted that the response patterns (see Figures 10B–F) and the highest Shepard tones (see Table 2) were not affected by the envelope center. The absolute predictions deviated from the data (*RMSD* = 0.17) because of the large deviation for the *C*_2_ envelope, where the threshold model predicted lower response rates of 0.5 for all tritone pairs (see Figure 10A). The prediction of the flat response function was due to the estimation of a very steep threshold function (see Figure 12), leading to a very high probability of all frequency components being filtered out for both Shepard tones and to an equal probability of the *higher* and *lower* responses (see Equation 19).

**Table 2:** Highest Shepard tones in the data predicted by the threshold model using logistic threshold functions for *PG*, who showed pronounced pitch class effects and AM, who showed pronounced envelope effects for the six envelope centers.

**Envelope**	***PG***		***AM***	
	**Data**	**Predicted**	**Data**	**Predicted**
*C*_2_	4.14 (D#)	11.70 (A#-B)	4.53 (D#-E)	4.51 (D#-E)
*C*_4_	5.78 (E-F)	5.57 (E-F)	7.27 (F#-G)	7.42 (F#-G)
*F#*_4_	5.46 (E-F)	5.34 (E-F)	12.49 (B-C)	12.34 (B-C)
*A*_4_	4.91 (E)	5.37 (E-F)	3.35 (D-D#)	2.93 (D)
*C*_5_	5.82 (E-F)	5.52 (E-F)	5.33 (E-F)	5.58 (E-F)
*D#*_6_	7.69 (F#-G)	7.72 (F#-G)	7.38 (F#-G)	7.58 (F#-G)

*Estimated logistic-threshold-function parameters for “PG” were â = 8.67, $\hat{b}=0.11$, $\hat{q}=0.29$, $\hat{l}=-0.57$, ${\hat{\sigma }}^{2}=0.27$ and for “AM” were â = 2.00, $\hat{b}=1.08$, $\hat{q}=0.50$, $\hat{l}=-12.02$, ${\hat{\sigma }}^{2}=0.12$*.

**Figure 11:**
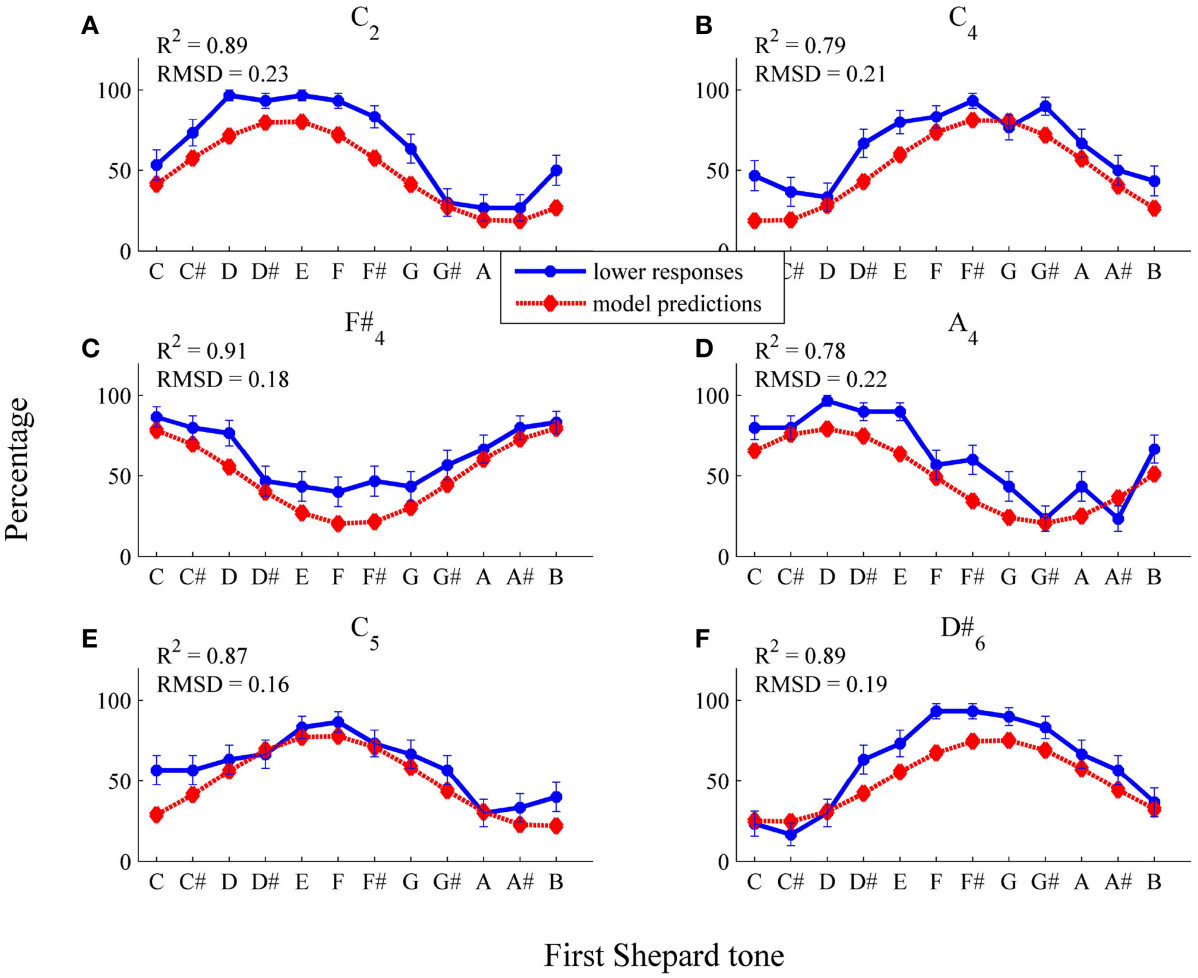
Fitted pronounced pitch class effects (participant AM): average percentages of *lower* responses (blue dots and unbroken line) in comparison to model predictions for the six envelopes **(A)** *C*_2_, **(B)** *C*_4_, **(C)** *F#*_4_, **(D)** *A*_4_, **(E)** *C*_5_, **(F)** *D#*_6_ (red diamonds and dotted line; estimated model parameters: â = 2.00, $\hat{b}=1.08$, $\hat{q}=0.50$, $\hat{l}=-12.02$, ${\hat{\sigma }}^{2}=0.12$).

As can be seen in Figure 11, the general trend of the responses of the participant AM (*R*^2^ = 0.89), who showed pronounced envelope effects (52.22%), was predicted well by the threshold model. The threshold model predicted that the response patterns (see Figure 11) and the highest Shepard tones (see Table 2) were affected by the envelope center. The absolute predictions deviated from the data (*RMSD* = 0.16), because the participant had an overall bias to give more *lower* responses (*M* = 62.45%), which is not implemented in the threshold model.

**Figure 12:**
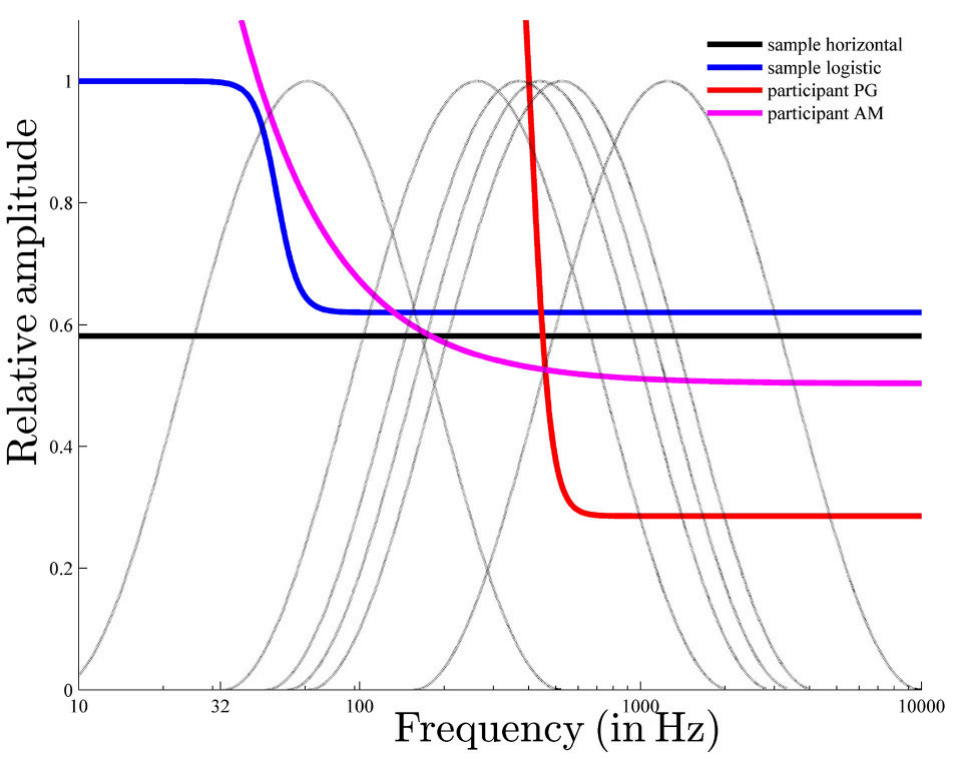
The six envelopes used in the experiment (gray lines) along with the estimated horizontal and logistic sample threshold functions and the individual logistic threshold functions for participants PG and AM, with pronounced pitch class and envelope effects, respectively. The estimated parameters of the horizontal sample threshold function were ${\hat{\mu }}_{t}=0.58$, ${\hat{\sigma }}^{2}=0.19$; that of the logistic sample were â = 5.67, $\hat{b}=0.13$, $\hat{q}=0.62$, *l* = 0, ${\hat{\sigma }}^{2}==0.18$; that of the logistic for “PG” were â = 8.67, $\hat{b}=0.11$, $\hat{q}=0.29$, $\hat{l}=-0.57$, ${\hat{\sigma }}^{2}=0.27$; and that of the logistic for “AM” were â = 2.00, $\hat{b}=1.08$, $\hat{q}=0.50$, $\hat{l}=-12.02$, ${\hat{\sigma }}^{2}=0.12$.

As can be seen in Figure 12, the threshold function used to predict pronounced pitch-class effects (the response pattern of *PG*) is much steeper than those used to predict pronounced envelope effects (the response pattern of *AM*). The steepness of the logistic function is determined by the parameter *b*. Possibly, this parameter determines the relationship between pitch class and envelope effects. Another important parameter is *a*, which determines the position of the logistic function on the frequency axis. Individual differences in this parameter, possibly, explain the individual differences among the highest pitch classes.

#### 3.2.3. Simulations

**Figure 13:**
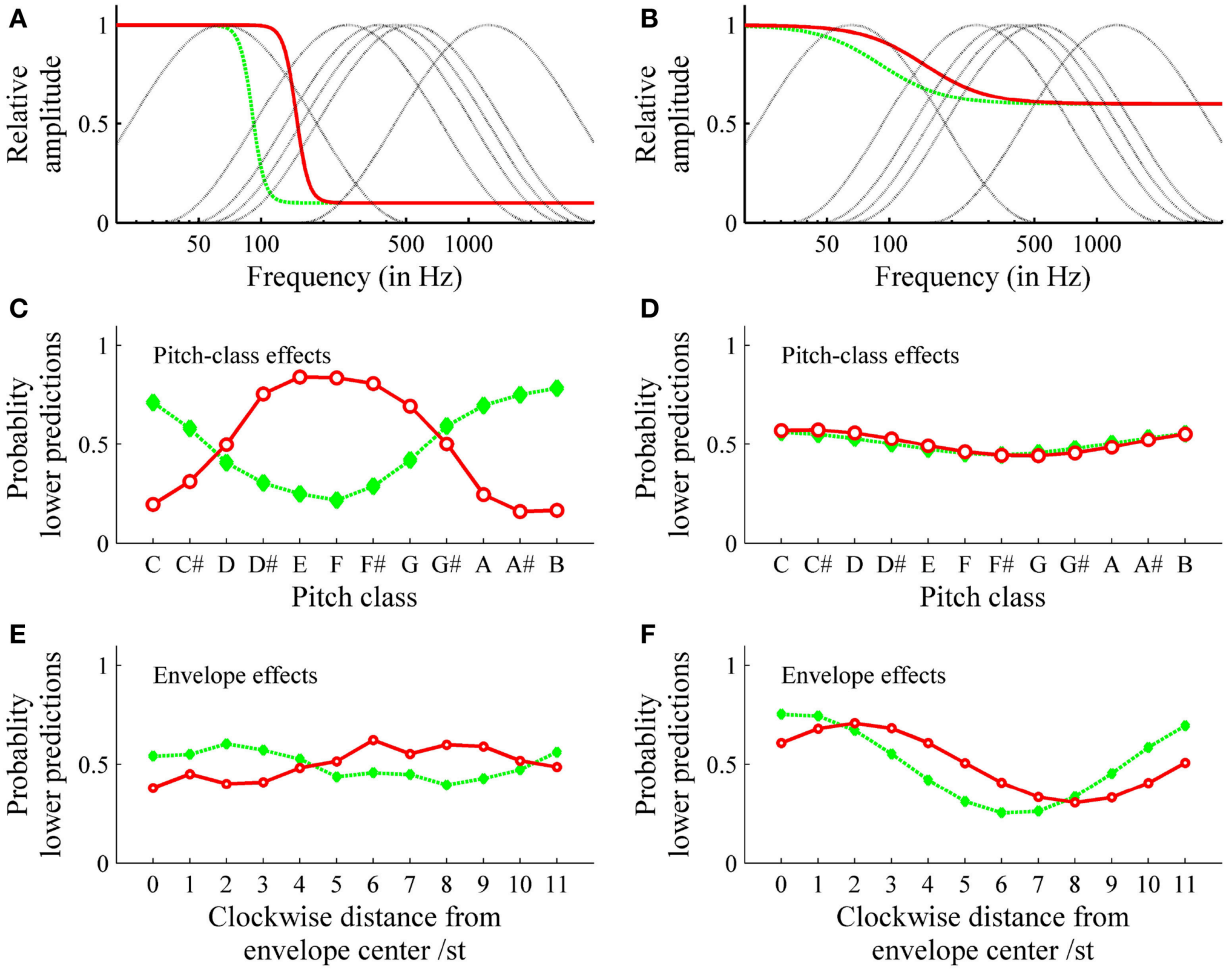
Simulation of pitch class **(C,D)** and envelope effects **(E,F)**: The steep (nearly vertical) threshold functions **(A)** cause pronounced pitch class **(C)** and small envelope effects **(E)**; shifting the threshold function on the frequency axis causes the shift in pitch class effects pattern in **(C)** (solid red or dashed green threshold functions lead to solid or dashed effects, respectively); the flat (nearly horizontal) threshold functions **(B)** cause small pitch class effects **(D)** and pronounced envelope effects **(F)**; shifting the threshold function on the frequency axis causes the shift in envelope effects pattern **(F)**. These simulations were performed for Shepard tones under the spectral envelopes used in the experiment (dotted line in **A**,**B**).

Simulations were conducted to investigate the effect of the form of the threshold function on the relationship between pitch class and envelope effects. The model input consisted of the logarithmic frequencies and the relative amplitudes of each tritone pair under each of the six envelopes that were used in the experiment (see Figures 13A,B). Pitch class and envelope effects were quantified using Repp's 1994 procedure as described above. Figure 13 shows that the threshold model predicted pitch class and envelope effects, depending on threshold function parameters. Particular effects depended on more than one parameter. However, by considering rather extreme threshold functions, some systematic associations were revealed: steep threshold functions caused pronounced pitch class (see Figure 13C) and small envelope effects (Figure 13E); that is, the highest pitch class did not depend on envelope centers. Shifting steep threshold functions on the frequency axis (see Figure 13A) caused nearly reversed patterns of pitch class effects, that is, opposite highest pitch classes (6 semitones removed; see Figure 13C). In contrast, flat threshold functions (see Figure 13B) caused pronounced envelope (Figure 13F) and small pitch class effects (see Figure 13D); that is, the highest pitch class did depend on the envelope center and was shifted by a particular distance from envelope center. Shifting flat threshold functions on the frequency axis (see Figure 13B) had no effect on pitch class effects (see Figure 13D) but had an effect on envelope effects (Figure 13F).

### 3.3. Discussion

The pitch-matching pretest revealed the octave ambiguity in pitch height of single Shepard tones. The further question was whether this ambiguity accounts for the tritone paradox, which would strengthen the psychoacoustic approach. The focus of the study was the introduction of a simple model that predicts responses to tone pairs by assuming the octave ambiguity in single Shepard tones. However, additionally, the study revealed further empirical evidence supporting the psychoacoustic approach.

The study replicated previous findings of an effect of the spectral envelope on the tritone paradox. The subjectively highest pitch classes were shifted about 5 semitones from the envelope center. Repp (1997) found shifts of about 6 semitones from the envelope center. The cause of this small deviation from the present study is unclear, but like in the Repp study, the Shepard tone that was nearly opposite to the envelope center was judged to be the highest. Thus, the spectral structure was the crucial factor in the present study. The finding of envelope effects supports the psychoacoustic approach, because, theoretically, changes in the spectral structure affect F0-extraction but not pitch classes.

Furthermore, the highest pitch class revealed in the pure tone matching task corresponded to that revealed in the tone-pair comparison task (pitch class *G* for *C*_4_ Shepard tones). In accordance with Repp and Thompson (2010), the highest Shepard tone assessable in the matching task corresponded to the highest Shepard tone of the tone-pair comparison task for averaged sample data.

The main finding of the study was that the threshold model, using a logistic threshold function, predicted the subjectively highest pitch classes, depending on the envelope center and the typical sigmoid response pattern for the sample and the individual data. Thus, in addition to the highest pitch class, the threshold model predicted that some tritone pairs were more ambiguous than other tritone pairs. Given that the threshold model implements the pitch ambiguity of single Shepard tones, the prediction of the typical sigmoid response pattern suggests that the octave ambiguity in single tones accounts for the tritone paradox.

The threshold model predicted the response patterns of participants who showed pronounced pitch class effects (highest pitch class is unaffected by the envelope center) and of those who showed pronounced envelope effects (the highest pitch class is affected by the envelope center). The estimated threshold function for the former was rather steep, while the estimated threshold function for the later was rather flat, indicating that the relationship between pitch class and envelope effects is mediated by the form of the threshold function.

Supporting this suggestion, the simulations showed that threshold functions that were mostly independent of frequency (horizontal or flat logistic threshold functions) account for small pitch class and pronounced envelope effects and that functions that are more closely dependent on frequency (steep logistic threshold functions) account for the reverse pattern. Furthermore, the simulation showed that the position of the threshold function on the frequency axis determines the highest pitch class.

Terhardt (1991) explained pitch class effects as a result of a frequency region where frequencies are especially salient (frequency preference region). Here, the VP (F0) extraction depends on this frequency preference region and only partly on the spectral structure of the Shepard tone. The findings of the present study add detail to this account. When participants possess a pronounced frequency preference region (implemented by steep threshold functions), that is, frequencies within a small frequency region are especially salient, pitch class effects are pronounced; however, when participants possess a wider frequency preference region (implemented by flat threshold functions), then envelope effects are pronounced. The position of the frequency preference region on the frequency axis (implemented by the position of the threshold function) determines the highest pitch class. Thus, the current approach supports and extends Terhardt (1991)'s explanation of the tritone paradox.

Two forms of threshold functions were tested: frequency-independent, horizontal and frequency-dependent, logistic threshold functions. The estimated logistic function was also nearly flat in the middle frequency region, possibly, because of the background noise. Zwicker and Feldtkeller (1967) showed that the hearing thresholds of pure tones, usually depending on their frequencies, become flat when background noise is present.

One cannot rule out that the finding of the current study that the tritone paradox was mainly affected by spectral factors and the finding of no common highest pitch class was due to the background noise. However, this result was also found in studies where no background noise was presented (Repp, 1994, 1997; Krüger and Lukas, 2002). Nevertheless, it would be interesting to test whether background noise affects pitch class and envelope effects, given that the threshold model predicts differences depending on flat or steep threshold functions.

Another topic to discuss is whether logistic functions are appropriate to approximate threshold functions. Possibly, the class of logistic functions is too restricted, and more complex threshold functions would be more appropriate. Trivially, more complex functions would improve fitting results by introducing more free parameters. The consequence, however, would be a more time-consuming fitting algorithm. Furthermore, the advantage of the logistic function is its simple form, which enables associations between specific function characteristics and specific effects in the tritone paradox, as revealed in the simulations.

Another aspect to discuss is whether the estimated threshold functions are in accordance with the general psychoacoustic parameters. For example, the parameter *a* can be interpreted as the lower limit of the frequency preference region. The estimate was 5.67 for the logistic threshold function in the sample, which is about 50 (2^5.67^ because of the logarithmic frequency scale) and corresponds, approximately, to the lower limit of the residual pitch (30–40 Hz; Ritsma, 1962; Moore, 1973; Krumbholz et al., 2000; Pressnitzer et al., 2001). It seems reasonable to look for additional associations between function characteristics and psychoacoustic or physiological parameters in future research. However, it would be more complicated to find associations between function parameters and effect characteristics for more complex threshold functions. Thus, rather simple functions seem preferable. Considering the advantages and disadvantages, the logistic function still seems to be an appropriate way to approximate threshold functions.

## 4. General discussion

The current study contributed to the theoretical discussion about the origin of the tritone paradox and strengthened the psychoacoustic explanation for Shepard tone phenomena. Even a very simplified pattern-matching model can explain the typical patterns found in the tritone paradox. Thus, a specific pitch class comparison mechanism postulated by Deutsch (1991) is not necessary to explain the tritone paradox. However, one cannot rule out that such a mechanism is at work in the tritone paradox, but owing to parsimony one should prefer the simpler, more general model.

One could argue that the threshold model only worked because mainly spectral factors affected the tritone paradox in the current study. However, the model also worked well for participants with pronounced pitch class and small envelope effects. Furthermore, the simulations showed that the threshold model predicts response patterns characterized by pronounced pitch class effects.

The current study contributes to the understanding of the tritone paradox by contributing further empirical data and by testing a theoretical model that is based on psychoacoustic assumptions. Whereas previous studies that support the psychoacoustic explanation of the tritone paradox focused on single Shepard tones, this study focused on comparisons of tone pairs. The typical response patterns of the tritone paradox were explained by considering Shepard tones as *normal* harmonic complex tones that are ambiguous with respect to their octave position.

One major drawback of the current study is that the individual data from the pitch-matching task and from the tone-pair comparison task (the standard tritone paradox) were not directly compared. Although such an approach seems reasonable and straightforward, it has several problems. Generally, individual data has the problem of individual factors (e.g., response biases), which can be eliminated, at least partly, by averaging across participants but not by averaging across trials within one participant. For example, some participants have an overall bias to give more *lower* than *higher* responses or *vice versa*. Repp and Thompson (2010) found no sufficient match between individual highest pitch classes assessed in their pitch-matching task and their tone comparison task, possibly caused by such methodological problems. Thus, the comparison of individual data is often less promising.

A problem of the threshold model is that it is hard to falsify, because it can be argued that a bad model fit is due to an inappropriate threshold function. More complex threshold functions with more free parameters necessarily improve the model fit. A possible solution would be to derive qualitative predictions from a specific form of threshold function. For example, assuming threshold functions to be logistic, the threshold functions are steeper within lower octaves and become flatter for higher octaves. Since steep threshold functions are associated with pronounced pitch class effects and flat threshold functions with pronounced envelope effects, more pronounced pitch class effects for lower octaves are expected than for higher octaves and the opposite pattern is expected for envelope effects.

A further limitation of the threshold model is that it is limited to tone pairs, ignoring the context effect shown in the literature (Englitz et al., 2013; Chambers and Pressnitzer, 2014; Chambers et al., 2017). The neuronal network model by Huang et al. (2015) predicts these context effects and, additionally, the sigmoid response pattern as a function of the pitch classes of tritone pairs without context. It is assumed that different Shepard tones correspond to different presynaptic strengths, affecting a synaptic weighting function. Such a weighting function corresponds to the threshold function implemented in the threshold model. In general, these models focus on different aspects of Shepard tone pitches. The neuronal network model focuses on the explanation of prior context effects, whereas the threshold model focuses on the explanation of pitch class and envelope effects.

The threshold model predicted a variety of ascending/descending patterns for tritone pairs, including patterns of pronounced pitch class and envelope effects and even more complex patterns, depending on the form of the threshold function. The threshold function can be interpreted as an internal spectral weighting function (Terhardt et al., 1982b) or a synaptic weighting function (Huang et al., 2015), with the result that a specific frequency region is particularly important for pitch. When such a preference region is clearly distinct, implemented through steep decreasing threshold functions, the threshold model predicts the typical findings of Deutsch (1991), that is, pronounced pitch class and small envelope effects. Under these conditions, the highest pitch classes are barely affected by envelope position and depend on the position of the preference region on the frequency axis (implemented by the position of the threshold function on the frequency axis). In contrast, when the preference region is less distinct, implemented through flat threshold functions, the threshold model predicts the findings of Repp (1994, 1997) or Krüger (2011), that is, pronounced envelope and small pitch class effects. Under these conditions, the highest pitch classes are largely affected by envelope center and do not depend on the position of the preference region anymore.

The threshold model leads to new suggestions about the connection between the tritone paradox and language. Remarkably, studies that revealed an effect of linguistic background also revealed pronounced pitch class and small envelope effects for most participants (Deutsch, 1991), whereas in studies that revealed no effect of linguistic background, most participants showed pronounced envelope effects or mixed patterns (Repp, 1994; Krüger and Lukas, 2002). Within the framework of the threshold model, the distinctness of the preference region determines the relationship between pitch class and envelope effects. Thus, the effects of linguistic backgrounds are, possibly, mediated by preference region distinctness. Deutsch, 1991 may have tested participants with distinct preference regions and, therefore, found a language connection, whereas Repp (1994) and Krüger and Lukas (2002) tested participants with less distinct preference regions and, therefore, found no language connection. In other words, to detect a connection between linguistic background and highest pitch classes, a sample of participants possessing distinct preference regions is required. Given that highest pitch classes depend on the position of the preference region, people of different linguistic backgrounds might differ in the position of the preference region. Perhaps specific frequency regions become especially sensitive during language development. The threshold model provides a potential link between language and the tritone paradox in the form of the position of the preference region on the frequency axis, which may explain the inconsistent findings regarding the influence of language on the paradox.

## Author contributions

The author confirms being the sole contributor of this work and approved it for publication.

### Conflict of interest statement

The author declares that the research was conducted in the absence of any commercial or financial relationships that could be construed as a potential conflict of interest.
