# Supplementary material for: Pitch Class and Envelope Effects in the Tritone Paradox Are Mediated by Differently Pronounced Frequency Preference Regions
Source: Front Psychol. 2018 Sep 28;9:1590. doi: 10.3389/fpsyg.2018.01590 (PMC6173142; doi:10.3389/fpsyg.2018.01590)
Supplement: Supplementary file 1 [file Appendix.pdf]

1 Appendix

1.1 Mathematical specification of the threshold model

Input. The threshold model is designed for spectral data, that is, for frequencies and amplitudes of the Shepard-tone components, instead of genuine sound stimuli. Model input consists of Shepard-tone pairs (s_l, s_k), in which the Shepard tone s_k follows the Shepard tone s_l. Both Shepard tones are different elements of the same stimulus set, s_l, $s_k \in \{s_1, \ldots, s_n\}$ and $l \neq k$. Each Shepard tone s_l is described by the sum of its m sinusoidal components for all times t:

$$s_l(t) = \sum_i^m a_{li} \cdot sin(2\pi f_{li} \cdot t - \phi_i), \tag{1}$$

with octave-spaced frequencies

$$\begin{aligned} f_{li} &= f_{l1} \cdot 2^{i-1}, \\ f_{l1} &= f_{\min} \cdot 2^{(l-1)/n} \end{aligned} \tag{2}$$

where $a_{li}, f_{li} > 0$. The amplitudes a_{li} are usually characterized by a cosine function. Each sinusoidal component c_{li} is characterized by the tuple $c_i = (f_i, a_i)$, called the i-th component of the Shepard tone s_l.[1] Hence, Shepard-tone pairs are characterized by

$$\begin{aligned} (s_l, s_k) &= \big(\{c_{l1}, \ldots, c_{lm}\}, \{c_{k1}, \ldots, c_{km}\}\big) \\ &= \big(\{(f_{l1}, a_{l1}), \ldots, (f_{lm}, a_{lm})\}, \{(f_{k1}, a_{k1}), \ldots, (f_{km}, a_{km})\}\big), \end{aligned} \tag{3}$$

for s_l, $s_k \in \{s_1, \ldots, s_n\}$ and $l \neq k$.

Both Shepard tones of a tone pair are assumed to be processed independently. Therefore, the following processing stages are described for single Shepard tones instead of tone pairs. Pitch processing for a single tone consists of extracting spectral pitches (stage I) and of extracting fundamentals (stage II).

Stage I. In the first stage, tones are decomposed into their sinusoidal components. The following assumptions are made:

1. All sinusoidal components are independently processed in different auditory filters. Consequently, each component triggers only the response of its own auditory filter.
2. An auditory filter only sends signals when the component amplitude is above a specific threshold value; otherwise it is filtered out. All nonfiltered components become spectral pitches, which are elements of the spectral-pitch pattern.

These assumptions are formalized below, including specific probability assumptions:

1. Each component c of the Shepard tone s is associated with the random variable V which codes whether the component is filtered out ($V = 0$) or not ($V = 1$).

[1] The phase ϕ_i is irrelevant for the model's predictions

2. If the Shepard tone s_l, characterized by the vector $(c_{l1},\ldots,c_{lm})$, is presented, then the random vector $\mathbf{V_l} = (V_{l1},\ldots,V_{lm})$ is realized. The realization $\mathbf{v_l} = (v_{l1},\ldots,v_{lm})$ is called the result-vector, resulting in $\mathbf{v_l} \in \{0,1\}^m$.

3. The probability $P(V_{li} = 1)$ is named p_{li}. Hence, the spectral pitch that corresponds to the component c_{li} exists with a probability of p_{li}.

4. The magnitude of the spectral pitch h^S_{li} is equal to the component frequency

$$h^S_{li} = f_{li}, \tag{4}$$

   and, trivially, $h^S_{li} \in \{f_{l1},\ldots,f_{lm}\}$.[2]

5. The set S_l represents the extracted spectral-pitch pattern of each Shepard tone s_l $(l = 1,\ldots,n)$ and is defined as

$$S_l := \{h^S_{li} | v_{li} = 1\}. \tag{5}$$

   It occurs with probability

$$P(\{S_l\}) = P(\{\mathbf{v_l}\}). \tag{6}$$

6. Due to the assumption of independent processing of the components, the probability of the result-vector $\mathbf{v_l} = (v_{l1},\ldots,v_{lm})$ is

$$P(\{\mathbf{v_l}\}) := P(\{(v_{l1},...,v_{lm})\}) = \prod_{i=1}^{m} P(\{v_{li}\}) = \prod_{i=1}^{m} p_{li}^{v_{li}} \cdot (1-p_{li})^{1-v_{li}}. \tag{7}$$

Until now, the probabilities p that components are not filtered out in the first processing stage were taken for granted. As mentioned before, the pitch processing of each Shepard tone s_l with m components c_{li} is considered as realizations of binary random variables V_{li}, which are Bernoulli-distributed with probabilities p_{li} $(l = 1,\ldots,n,$ $i = 1,\ldots,m)$, where each random variable V_{li} indicates whether or not the component c_{li} is filtered out. To calculate probability p_{li}, the conditions under which the component c_{li} is filtered out (or not) have to be specified. The component c_{li} is assumed to not be filtered out if its amplitude a_{li} reaches the threshold value t_{li}, which is normally distributed according to the classical threshold theory (Gescheider, 1997). Thus, for components c_{li}

$$T_{li} \sim N(\mu_t, \sigma^2), \tag{8}$$

for all $l = 1,\ldots,n$, $i = 1,\ldots,m$. Accordingly, the threshold value t_{li} is a realization of the random variable T_{li}. The probability p_{li} that the component c_{li} is not filtered out is defined by the probability that its amplitude a_{li} is greater than the threshold value t_{li}. This probability is defined by the area under the probability density function of T_{li} for $t \leq a_{li}$. Thus,

$$p_{li} := P(T_{li} \leq a_{li}) = F_{li}(a_{li}), \tag{9}$$

[2]Physical units are disregarded within the threshold model.

where F_{li} is the distribution function of T_{li} and $l = 1,\dots,n$, $i = 1,\dots,m$. Thus, the probability that a component is not filtered is the area under the probability density function. The expected value μ_t of the random variable T_{li} is determined by the threshold function g, which is estimated from the empirical data and can depend on the frequencies f_{li}.

Stage II. In the second stage, each spectral-pitch pattern is matched to a fundamental, which is dedicated to the lowest element in the spectral-pitch pattern S. This suggestion is formalized in the following, including again probability assumptions:

1. Every set S_l is assigned to the fundamental $f0_l$, where

$$f0_l := \min(S_l) \tag{10}$$

   and, trivially, $f0_l \in \{h^S_{l1},\dots,h^S_{lm}\}$ and, hence, $f0_l \in \{f_{l1},\dots,f_{lm}\}$.

2. The probability q_{li} is the probability of the set S_l, such that $f0_l = h^S_{li}$ with $i = 1,\dots,m$.

3. This term holds true only when the random vector $\mathbf{V_l}$ is realized so that the i-th component is the lowest nonfiltered component ($v_{li} = 1$, $v_{lj} = 0$ for all $j < i$), hence for $l = 1,\dots,n$ the probability

$$\begin{aligned} P(f0_l = h^S_{li}) := \quad & q_{li} = \\ = \quad & P(\{(v_{l1},\dots,v_{lm}) | v_{li} = 1, v_{lj} = 0, \text{for all } j < i\}). \end{aligned} \tag{11}$$

   Applying equation 7, the probabilities q_{li} are calculated as follows (for $i = 1,\dots,m$, $l = 1,\dots,L$):

$$q_{li} = \begin{cases} \text{for } i = 1: & p_{l1} \\ \text{for } i > 1: & p_{li} \cdot \prod_{s=1}^{i-1}(1 - p_{ls}). \end{cases} \tag{12}$$

4. When all components are filtered out, a fundamental cannot be determined. The probability is then q_{l0} and is

$$q_{l0} = \prod_{s=1}^{m}(1 - p_{ls}), \tag{13}$$

according to Equation 7. At the end of stage II, a pattern of fundamental estimates emerges, referring to probability density functions.

Stage III. The input of stage III consists of the stage-two output of the Shepard tone s_l and the stage-two output of the Shepard tone s_k (s_l, $s_k \in \{s_1,...,s_n\}$, $l \neq k$). The aim is to obtain the probabilities of descending judgments. A descending judgment is defined as occurring when the fundamentals of the first tone are higher than the fundamentals of the second tone. This event occurs when the lowest first-tone spectral pitch is higher than the lowest second-tone spectral pitch. This suggestion is now formalized as follows:

1. When the realizations of the random vectors $\mathbf{V_l}$ and $\mathbf{V_k}$ are $f0_l = h^S_{li}$ and $f0_k = h^S_{kj}$, $f0_l > f0_k$ only if $h^S_{li} > h^S_{kj}$.

2. The probability o_{ij} that the joint event of $f0_l = h^S_{li}$ and $f0_k = h^S_{kj}$ occurs is defined, assuming independence of the 2 tones, as follows:

$$\begin{aligned} P(f0_l = h^S_{li}, f0_k = h^S_{kj}) := & \quad o_{ij} = \\ = & \quad P(f0_l = h^S_{li}) \cdot P(f0_k = h^S_{kj}) = \\ = & \quad q_{li} \cdot q_{kj}. \end{aligned} \tag{14}$$

3. The probability of descending judgments $P(s_l \succ s_k)$ for the tone pair (s_l, s_k) comprises the sum of probabilities $P(f0_l = h^S_{li}, f0_k = h^S_{kj})$ with $h^S_{li} > h^S_{kj}$ and the halved (thus far ignored) probabilities r_{lk} that all components of the first or all components of the second Shepard tone are filtered out, in other words, that $\mathbf{v_l} = (v_{li} | v_{li} = 0)$ or $\mathbf{v_k} = (v_{kj} | v_{kj} = 0)$ is realized. When everything is filtered out, then it is assumed that listeners guess *higher* or *lower* with equal probability. Thus,

$$P(s_l \succ s_k) := \sum_{i,j | h^S_{li} > h^S_{kj}} o_{ij} + 0.5 \cdot r_{lk}, \tag{15}$$

where

$$\begin{aligned} r_{lk} = & q_{l0} + q_{k0} - q_{l0} \cdot q_{k0}, \\ r_{lk} = & \prod_{i=1}^{m} (1 - p_{li}) + \prod_{j=1}^{m} (1 - p_{kj}) - \prod_{i=1}^{m} (1 - p_{li}) \cdot \prod_{j=1}^{m} (1 - p_{kj}). \end{aligned} \tag{16}$$

To calculate the probabilities $P(s_l \succ s_k)$ in Equation 15, the indices i and j are needed so that $h^S_{li} > h^S_{kj}$. Given that $h^S_{li} = f_{li}$ and $h^S_{kj} = f_{kj}$ (compare Eq. 4), these required indices can be derived from the arrangement of the frequencies f_{li} and f_{kj}. The frequencies f_{li} and f_{ki} can be alternately arranged according to their size, which means that for the Shepard-tone pair (s_l, s_k) with $l > k$, each frequency of the y-th component of s_l is higher than the frequency of the y-th component of s_k and for a Shepard-tone pair (s_l, s_k) with $l < k$ the opposite is true. Hence, for all $y = 1, \ldots, m$ the following is true (see below for a proof):

$$l > k : f_{ly} > f_{ky}, \tag{17}$$

$$l < k : f_{ly} < f_{ky}. \tag{18}$$

Thus,

$$f_{li} > f_{kj} \text{ if } \begin{cases} i \geq j, & \text{for } l > k \\ i > j, & \text{for } l < k. \end{cases} \tag{19}$$

Thus,

$$\begin{aligned} P(s_l \succ s_k) = & \sum_{i=1}^{m} \sum_{j=a}^{m} o_{ij} + 0.5 \cdot r_{lk} \\ = & \sum_{i=1}^{m} \sum_{j=a}^{m} (q_{li} \cdot q_{kj}) + 0.5 \cdot r_{lk}, \end{aligned} \tag{20}$$

with

$$a = \begin{cases} i, & \text{for } l > k, \\ i+1, & \text{for } l < k. \end{cases} \tag{21}$$

1.2 Justification of model assumptions

1. The assumption that all sinusoidal components are independently processed in different auditory filters is reasonable for Shepard tones on the basis of physiological data and the special construction of Shepard tones. Commonly, the cochlea is considered as a bank of parallel, overlapping auditory band-pass filters characterized by center frequencies and bandwidths. The bandwidths are commonly estimated by equivalent rectangular bandwidths (ERBs, Glasberg & Moore, 1990), approximated as the function of the auditory filter's center frequency f_c by the following equation:

$$ERB_{f_c} = 24.7(0.00437 \cdot f_c + 1). \tag{22}$$

Components are considered to be processed in different auditory filters if they are resolved (Cheveigné, 2010), which occurs if components of a complex tone can be "heard out" with a distinct pitch (e.g., Moore & Ohgushi, 1993; Plomp, 1964; Plomp & Mimpen, 1968). Moore and Ohgushi (1993) found this hearing out for adjacent frequencies that differ by about 1.25 ERBs from each other. Thus, when adjacent frequencies differ by more than 1.25 ERBs, it seems reasonable to assume that the Shepard-tone components c_i can be resolved, which holds true if the following equation is true:

$$f_i + 1.25 \cdot ERB_{f_i} < f_{i+1} \tag{23}$$

for $i \in \mathbb{N}$. For Shepard tones consisting of m components, $f_{i+1} = 2 \cdot f_i$ for all $i = 1, \ldots, m-1$ because of the octave-spaced components. Applying this expression to Equation 23 results in

$$f_i + 1.25 \cdot ERB_{f_i} < 2 \cdot f_i. \tag{24}$$

Substituting Equation 22 into Equation 24, the following equation results

$$f_i + 1.25 \cdot [24.7(0.00437 \cdot f_i + 1)] < 2 \cdot f_i, \tag{25}$$

which simplifies to

$$f_i > 35.69. \tag{26}$$

Equation 26 indicates that frequency components higher than 35.69 Hz are separated by at least 1.25 ERB from their adjacent frequency components and are therefore considered resolved. However, given that frequency components lower than 35.69 Hz are attenuated by the Shepard-tone envelope and additionally lie next to the lower limit of pitch (30 - 40 Hz; Krumbholz, Patterson, & Pressnitzer, 2000; Moore, 1973; Pressnitzer, Patterson, & Krumbholz, 2001; Ritsma,

1962; Zwicker & Feldtkeller, 1967), the assumption that all Shepard-tone components can be resolved and, hence, are processed in different auditory filters is reasonable.
The assumption that components in different auditory filters are processed independently is supported by Zwicker and Feldtkeller (1967). They revealed that the sound intensities of two pure tones are only summed for further processing if their frequencies lie within one auditory filter. Furthermore, information in different auditory filters does not interact . Likewise, recognition of vowels (Apoux & Healy, 2009) and temporal gap detection (Phillips & Hall, 2000) were also nearly unaffected by nonoverlapping noise bands. Additionally, Shackleton and Carlyon (1994) found that the relation between component phases has no effect on pitch for resolved components. In sum, the reasonable assumptions can be made that Shepard-tone components are characterized only by their frequencies and amplitudes and are processed independently in different auditory filters.

2. The assumption that an auditory filter only sends signals when the component amplitude is above a specific threshold value is consistent with the physiological response-triggering of action potentials when the depolarization of receptors (inner hair cells) exceeds a threshold value (all-or-none law; Hodgkin & Huxley, 1945; Hudspeth, 1985). Stevens, Morgan, and Volkmann (1941) showed that such neural processes are in accordance with the assumptions of the classical threshold theory (Gescheider, 1997).

3. The fundamental frequency is defined as to the frequency of the lowest element in the spectral-pitch pattern. In general, frequency components f_i of harmonic-complex tones are integer multiples of the fundamental $f0$ ($f_i = i \cdot f_0$). Thus, this assumption is reasonable when each frequency component is an integer multiple of each lower-frequent component (because, in general, $f_i = i \cdot f_0$). Thus, it can be shown that for all Shepard tones s_l with frequency f_{li} ($l = 1, \ldots, n$, $i = 1, \ldots, m$), $f_{li} = f_{l(i-k)} \cdot j$, where $j \in \mathbb{N}_{<0}$ is true for $k < i, k \in \mathbb{N}_{<0}$. Applying the definition of frequencies f_{li}, given in Equation 2, to this expression, the following equation results in
$$f_{l1} \cdot 2^{(i-1)} = j \cdot (f_{l1} \cdot 2^{i-k-1}), \quad (27)$$
which simplifies to
$$j = 2^k. \quad (28)$$
Because $i, k \in \mathbb{N}_{<0}$ and $k < i$ also $j \in \mathbb{N}_{<0}$. □

Proof. It can be shown that for all Shepard-tone pairs (s_l, s_k) with $l < k$, $f_{li} < f_{ki}$ and with $l > k$, $f_{li} > f_{ki}$ is true ($l, k = 1, \ldots, n, l \neq k, i = 1, \ldots, m$). For Shepard tone s_l the following is true because of Equation 2:

$$f_{li} = f_{\min} \cdot 2^{[(i-1) \cdot L + (l-1)]/L} \quad (29)$$

So the following is valid:

$$
\begin{aligned}
f_{li} &< f_{ki} \\
f_{\min} \cdot 2^{[(i-1)\cdot n+(l-1)]/n} &< f_{\min} \cdot 2^{[(i-1)\cdot n+(k-1)]/n} \\
\frac{f_{\min} \cdot 2^{[(i-1)\cdot n+(l-1)]/n}}{f_{\min} \cdot 2^{[(i-1)\cdot n+(k-1)]/n}} &< 1 \\
2^{l-k} &< 1 \\
l-k<0 & \\
l<k & \\
q.e.d. &
\end{aligned}
\tag{30}
$$

Also true is:

$$
\begin{aligned}
f_{li} &> f_{ki} \\
f_{\min} \cdot 2^{[(i-1)\cdot L+(l-1)]/L} &> f_{\min} \cdot 2^{[(i-1)\cdot L+(k-1)]/L} \\
l-k>0 & \\
l>k & \\
q.e.d. &
\end{aligned}
\tag{31}
$$

References

Apoux, F., & Healy, E. W. (2009). On the number of auditory filters outputs needed to understand speech: Further evidence for auditory channel independence. *Hearing Reseach*, *255'(1-2)*, 99-108. doi: 10.1016/j.heares.2009.06.005

Cheveigné, A. d. (2010). Pitch perception. In C. J. Plack (Ed.), *The oxford handbook of auditory science: Hearing* (pp. 71–104). Oxford: University Press.

Gescheider, A., George. (1997). *Psychophysics. the fundamentals* (Third Edition. ed.). Mahwah, New Jersey: Lawrence Erlbaum Assciates.

Glasberg, B. R., & Moore, B. C. J. (1990). Derivation of auditory filter shapes from notched-noise data. *Hearing Reseach*, *47*, 103–138. doi: 10.1016/0378-5955(90)90170-T

Hodgkin, A., & Huxley, A. (1945). Resting and action potentials in single nerve fibres. *The Journal of physiology*, *104*(2), 176–195. Retrieved from `http://onlinelibrary.wiley.com/doi/10.1113/jphysiol.1945.sp004114/pdf`

Hudspeth, A. (1985). The cellular basis of hearing: the biophysics of hair cells. *Science*, *230*(4727), 745-752. doi: 10.1126/science.2414845

Krumbholz, K., Patterson, R. D., & Pressnitzer, D. (2000, Sep). The lower limit of pitch as determined by rate discrimination. *Journal of Acoustical Society of America*, *108*(3 Pt 1), 1170–1180. doi: 10.1121/1.1287843

Moore, B. C. J. (1973). Some experiments relating to the perception of complex tones. *Quarterly Journal of Experimental Psychology*, *25*(4), 451–475. doi: 10.1080/14640747308400369

Moore, B. C. J., & Ohgushi, K. (1993). Audibility of partials in inharmonic complex tones. *Journal of Acoustical Society of America*, *93*, 452–461. doi: 10.1121/1.405625

Phillips, D. P., & Hall, S. E. (2000). Independence of frequency channels in auditory temporal gap detection. *The Journal of the Acoustical Society of America*, *108*(6), 2957–2963.

Plomp, R. (1964). The ear as a frequency analyzer. *Journal of the Acoustical Society of America*, *36*, 1628–1636. doi: 10.1121/1.1919256

Plomp, R., & Mimpen, A. M. (1968). The ear as a frequency analyszer ii. *Journal of Acoustical Society of America*, *43*, 764–767. doi: 10.1121/1.1910894

Pressnitzer, D., Patterson, R. D., & Krumbholz, K. (2001). The lower limit of melodic pitch. *Journal of Acoustical Society of America*, *109*(5 Pt 1), 2074–2084. doi: 10.1121/1.1359797

Ritsma, R. J. (1962). Existence region of the tonal residue. *Journal of Acoustical Society of America*, *34(9)*, 1224–1229. doi: 10.1121/1.1918307

Shackleton, T. M., & Carlyon, R. P. (1994, Jun). The role of resolved and unresolved harmonics in pitch perception and frequency modulation discrimination. *Journal of Acoustical Society of America*, *95*(6), 3529–3540. doi: 10.1121/1.409970

Stevens, S. S., Morgan, C., & Volkmann, J. (1941). Theory of the neural quantum in the discrimination of loudness and pitch. *The American Journal of Psychology*, *54*(3), 315–335.

Zwicker, E., & Feldtkeller, R. (1967). *Das Ohr als Nachrichtenempfänger*. Stuttgart: Hirzel Verlag.
